# Construction of a SSR-Based Genetic Map and Identification of QTLs for Catechins Content in Tea Plant (*Camellia sinensis*)

**DOI:** 10.1371/journal.pone.0093131

**Published:** 2014-03-27

**Authors:** Jian-Qiang Ma, Ming-Zhe Yao, Chun-Lei Ma, Xin-Chao Wang, Ji-Qiang Jin, Xue-Min Wang, Liang Chen

**Affiliations:** Key Laboratory of Tea Plant Biology and Resources Utilization, Ministry of Agriculture, National Center for Tea Improvement, Tea Research Institute of the Chinese Academy of Agricultural Sciences (TRICAAS), Hangzhou, Zhejiang Province, China; New Mexico State University, United States of America

## Abstract

Catechins are the most important bioactive compounds in tea, and have been demonstrated to possess a wide variety of pharmacological activities. To characterize quantitative trait loci (QTLs) for catechins content in the tender shoots of tea plant, we constructed a moderately saturated genetic map using 406 simple sequence repeat (SSR) markers, based on a pseudo-testcross population of 183 individuals derived from an intraspecific cross of two *Camellia sinensis* varieties with diverse catechins composition. The map consisted of fifteen linkage groups (LGs), corresponding to the haploid chromosome number of tea plant (2n = 2x = 30). The total map length was 1,143.5 cM, with an average locus spacing of 2.9 cM. A total of 25 QTLs associated with catechins content were identified over two measurement years. Of these, nine stable QTLs were validated across years, and clustered into four main chromosome regions on LG03, LG11, LG12 and LG15. The population variability explained by each QTL was predominantly at moderate-to-high levels and ranged from 2.4% to 71.0%, with an average of 17.7%. The total number of QTL for each trait varied from four to eight, while the total population variability explained by all QTLs for a trait ranged between 38.4% and 79.7%. This is the first report on the identification of QTL for catechins content in tea plant. The results of this study provide a foundation for further cloning and functional characterization of catechin QTLs for utilization in improvement of tea plant.

## Introduction

Tea, one of the most widely consumed non-alcoholic beverages in the world, is an infusion of the cured leaves of tea plant (*Camellia sinensis* (L.) O. Kuntze), which is grown primarily in the tropical and subtropical regions of Asia, Africa, and Latin America. According to the International Tea Committee (ITC), over 4.21 million tons of made tea were produced in 2011, of which more than 84% came from Asia [1]. The number of tea plant grown in China, the world's largest tea producer, has increased to around 2.11 million hectares with an annual production of about 1.62 million tons, contributing nearly 37.8% of the world production, followed by India (23.0%), Kenya (8.8%), Sri Lanka (7.6%), and Vietnam (4.1%) [1].

Tea recently has attracted worldwide attention for its multiple health-promoting effects, especially in regard to its potential for chemoprevention of cancer, cardiovascular, and neurological diseases [2]–[5]. Increasing scientific and customer interest in the health benefits of tea has led to the increased consumption of tea products, particularly with respect to tea extracts, which can be used as a featured ingredient in a range of food, beverage, and cosmetic products [6], [7]. Tea contains a number of bioactive compounds, such as the flavonoids, caffeine, and L-theanine. Overall, the major constituents in tea extracts are catechins, a group of flavonoids, which contribute up to 30% of the dry weight of the tender tea shoots (“two and a bud”, i.e. one apical bud and two adjacent leaves) [8]. Catechins in green tea leaves consist mainly of four flavan-3-ols, viz. (-)-epicatechin (EC), (-)-epicatechin gallate (ECG), (-)-epigallocatechin (EGC), and (-)-epigallocatechin gallate (EGCG) [9]. Of these, EGCG is the most abundant, accounting for up to 70% of the total tea catechins [9], and have been demonstrated to possess a wide variety of pharmacological activities including antioxidant and radical-scavenging activity, which is thought to be an underlying protective mechanism primarily responsible for the health benefits of tea [2], [10], [11]. Therefore, the issue of improving catechins content and composition has become increasingly important in tea plant breeding programs.

The tea plant is a dicotyledonous, perennial, and woody plant. Its diploid genome consists of 15 chromosome pairs, with an estimated size of ∼4.0 Giga bases [12]. There are two main cultivated varieties of tea plant, i.e. the *C.sinensis* var. *sinensis*, which is predominantly grown in China, Japan and Turkey, where most of the harvest is used to produce green tea, and the *C.sinensis* var. *assamica* (Masters) Kitamura, which is cultivated worldwide and usually used for black tea production due to its high content of tea polyphenols. The tea plant is highly heterozygous, generally self-incompatible, and its juvenile stage can span up to 4–5 years. Traditional approaches for genetic improvement in tea plant is therefore extremely difficult and time-consuming, especially for complex traits such as catechins content, which are controlled by multiple genes known as quantitative trait loci (QTLs).

Linkage-based QTL mapping is a powerful tool for elucidating the molecular basis of complex traits, facilitating identification of specific genes responsible for particular genetic traits and estimation of gene actions and genetic parameters. And it can also provide useful insight into the development of new approaches to improve the efficiency and precision of conventional plant breeding via marker-assisted selection (MAS) [13], [14]. A number of QTLs for important agronomic traits have been detected and characterized in grain crops (e.g. rice [15], maize [16]), vegetables and fruits (e.g. tomato [17], eggplant [18]). However, the application of QTL mapping in tea plant is difficult. One reason is that the natural features of self-incompatibility and low seed yield of tea plant make it difficult to create suitable mapping populations, and another one is the lack of available genetic markers. To the best of our knowledge, there have been only seven reported cases of linkage maps and one case of mapping QTLs of yield related traits in tea plant [19]–[26]. The catechins content, though being major determinants of tea quality, have not yet been subjected to QTL analysis.

SSR, also known as microsatellite, is one of the most popular and versatile marker type used in plant genetic mapping studies, due to its desirable features such as high abundance, locus specificity, codominant inheritance, high polymorphism information content, and reproducibility [27]. According to the origins, SSRs are divided into two categories: genomic SSRs or genic SSRs (EST-SSRs). The development of genomic SSR markers has traditionally been a difficult, costly, and time-consuming process, which involves the construction of genomic library enriched for specific repeat motifs [28]. To date, only a limited number of validated genomic SSR markers (<100) are available for tea plant [29]–[33]. In contrast, the genic SSRs can easily be developed by screening the collection of clustered ESTs in publicly available databases. Furthermore, the genic SSRs are derived from the expressed regions of the genome, and thus have increased potentials for tagging and mapping of genes and QTLs [27]. The genic SSRs are also highly transferable, because their flanking sequences are more likely to be conserved in related species, and therefore can be used as anchor markers for comparative mapping. The approximate total number of genic SSR markers available in tea plant has been increased to 900 thus far [25], [34]–[43]. However, it is still insufficient for constructing a saturated SSR-based genetic map of tea plant.

Recently, we obtained approximately 57 million RNA-Seq reads by deep sequencing of the tea plant transcriptome, using the Illumina sequencing platform [44]. These sequence data provide a good resource for the development of genic SSR markers. Thus the aim of the current study were to: (1) develop a set of novel genic SSR markers for tea plant, (2) construct a SSR-based genetic linkage map using a pseudo-testcross population generated from an inter-varietal cross of *C. sinensis*, and (3) identify QTLs controlling catechins content in the tender shoots for the marker assisted selection of tea plant in the near future.

## Materials and Methods

### Mapping population and DNA isolation

A pseudo-testcross population consisting of 300 individuals was developed by crossing two tea plant accessions chosen from the China National Germplasm Tea Repository (CNGTR) in the TRICAAS located at Hangzhou, Zhejiang, China. The maternal parent, ‘Yingshuang’ (hereinafter ‘YS’), is an improved cultivar derived from the cross of two major Chinese cultivar, *C. sinensis* var. *sinensis* cv. *Fuding Dabaicha* (hereinafter ‘FD’) and *C. sinensis* var. *assamica* cv. *Yunnan Dayecha* (hereinafter ‘YD’). The paternal parent, ‘Beiyue Danzhu’ (hereinafter ‘BD’), is a landrace of *C. sinensis* var. *pubilimba* Chang originally collected from Southwest China. The cultivar ‘YS’ has an early-sprouting growth habit, medium-sized leaves, and moderate catechins content in the tender shoots, as compared to ‘BD’ which has a late-sprouting growth habit, large-sized leaves, and higher catechins content in the tender shoots. The entire population and both parents were grown at the TRICAAS Experimental Station. A subset of 183 individuals was selected for genetic mapping and QTL analysis. DNA was extracted from young leaves and buds of each plant using a CTAB method with minor modifications [45]. DNA quality was assessed by electrophoresing in a 0.8% TBE-agarose gel, stained with ethidium bromide. DNA concentration was determined utilizing UV/Vis spectroscopy, and then adjusted to 10 ng/mL for SSR analysis.

### SSR mining and primer design

A set of unigene sequences generated from the transcriptome assembly of tea plant in our previous studies [44] were searched for SSRs using the MISA software (http://pgrc.ipk-gatersleben.de/misa; [46]). For di-, tri-, tetra-, penta-, hexa- and higher-order nucleotide motifs, the minimum numbers of repeats were defined as 8, 5, 4, 3, 3 and 3, respectively. Primer pairs were designed based on the flanking sequences of each SSR using Primer3 (http://bioinfo.ut.ee/primer3-0.4.0/primer3/) with threshold criteria of 18–24 bp primer length, 40–60% GC content, and an estimated amplicon size of 100–300 bp.

### SSR screening and genotyping

A total of 1,509 SSR primer pairs, including 1,141 newly developed and 368 previously reported in different *Camellia* species, were initially screened for polymorphism between the mapping parents. Those primer pairs that successfully amplified a single locus, which was polymorphic in at least one of the parents, were subsequently used to determine the genotype of F_1_ progeny. PCR mixture consisted of the following in 10 µL total volume: 10 ng genomic DNA, 10 mM dNTPs, 10 µM of each primer, 0.5 U Taq polymerase (Beijing Dingguo Biotech, Beijing, China) and 10× PCR buffer supplied together with the enzyme. Amplification was performed according to the method of Zhao et al. [35] using the following reaction conditions: 4 min at 94°C for initial denaturation, followed by 35 cycles of 94°C for 30 s, annealing temperature for 30 s, 72°C for 30 s, with a final extension at 72°C for 7 min. PCR products were separated in 10% polyacrylamide gels and detected by silver staining [47].

### Linkage map construction

The genetic map was constructed using a pseudo-testcross mapping strategy as described by Grattapaglia and Sederoff [48]. Linkage analyses were performed using the JoinMap 4 software with a cross-pollinator (CP) population type [49]. Chi-square statistics were calculated for each marker to assess the segregation deviation (SD) from the expected Mendelian ratios. Both distorted and non-distorted markers were used for linkage mapping. A framework linkage map was primarily constructed based on non-distorted markers, using a “One-step method” as described by Rabbi et al. [50]. In brief, a logarithm of odds ratio (LOD) score of 4.0 was used to assign markers to linkage groups (LGs), in which the ordering of markers was determined using regression mapping with default parameters. Then, the distorted markers were separately added onto the linkage map, except the ones greatly affecting the order of their neighbor markers or excessively changing linkage distance. The Kosambi function was used for the estimation of map distances. The graphical maps were generated using MapChart version 2.1 [51].

### Catechins extraction and identification

In the spring of 2010 and 2011, the “two and a bud” tender shoots for each plant were immediately collected and steamed at 120°C for 5 min, and then dried to constant weight at 80°C. The isolation and detection of tea catechins, by high performance liquid chromatography (HPLC), was performed according to the International Standards Organization (ISO) ISO 14502-2-2005(E) procedure [52] with minor modifications. In brief, the processed tea shoots were ground into a fine powder, and a sample of 0.2±0.0001 g of the powder was subsequently extracted twice with 5 mL of 70% methanol by heating at 70°C in water bath for 10 min, followed by centrifuging at 3500 rpm for 10 min. The extracts were combined and made up to 10 mL with 70% methanol, and then 1 mL volumes of the extracts were diluted to 5 mL with stabilizaing solution (10% v/v acetonitrile, 500 µg/mL EDTA and ascorbic acid). Afterward the dilutions were filtered through 0.45 µm nylon filters, and 10 µL were applied to HPLC-UVD (Waters 2695-2489) for catechins quantification. HPLC was carried out on a SunFire C_18_ column (5 µm, 4.6×250 mm) at 35°C. A binary gradient elution system was adopt with mobile phase consisting of solvent A (1% v/v formic acid) and solvent B (acetonitrile). The elution profile was as follows: 93.5 to 85% A from 0 to 16 min, 85 to 75% A from 16 to 20 min, 75.0 to 93.5% A from 20 to 26 min, and held at 93.5% from 26 to 30 min to equilibrate the column for the next injection. The flow rate was maintained at 1 mL/min, and the detection wavelength was set at 278 nm. Individual catechins were identified based on retention times of unknown peaks in comparison with those of peaks obtained from catechin standards, and the calibration curves for quantification were generated for each catechin by linear regression of the standard peak areas versus the standard concentrations in mg/mL (Figure S1).

### Statistical analysis and QTL mapping

Statistical analysis of phenotypic data was conducted using the SAS software (SAS Institute Inc., Cary, North Carolina). For each trait, the mean and standard deviation of each plant was calculated, and the significance of differences between parental values was analyzed using the Mann-Whitney U test. Pearson's correlation coefficients for pairwise trait combinations were also calculated. QTL mapping was performed using the MapQTL 4.0 software, employing the single-QTL model (i.e. interval mapping, IM) in combination with the restricted multiple QTL model (rMQM) [53]. In brief, the IM was performed to detect QTLs, and then the rMQM was used to refine QTL positions. In rMQM mapping, the marker with the highest LOD score was selected as a cofactor, and multiple rounds of rMQM were performed until the cofactors selected were stable. The LOD score threshold for QTL declaration was determined using the permutation test (1,000 replications) at a genome-wide level of 5%. QTL were designated for peaks that reached significance, and QTL regions were 1- and 2-LOD support intervals. QTL for each trait across different years were declared to be stable if their confidence intervals overlapped. A QTL was classified as major/minor based on 10% of explained population variability (PVE). The additive (or average allele substitution) effects from maternal parent ‘YS’ (a1) and paternal parent ‘BD’ (a2), and dominance effects of each QTL were estimated by the model of Knott et al. [54] as used by Qin et al. [55]. The maternal parent ‘YS’ was derived from the cross of ‘FD’ and ‘YD’, therefore the QTL with a positive and negative a1 meant that the allele for increasing catechins content was contributed by ‘FD’ and ‘YD’, respectively. The a2 was not further analyzed due to a lack of required information about the parents of ‘BD’.

## Results

### Novel genic SSRs

A total of 59,962 unigenes derived from the transcriptome assembly of tea plant were randomly selected and used for SSR mining. The results showed that there were overall 7,589 SSR-contained unigenes, representing a total number of 9,239 genic SSRs. The estimated distance between SSRs was 3.98 kb on average, corresponding to one SSR for every 12.7 unigenes. Dinucleotide was the most common repeat unit (38.9%), followed by tri- (27.2%), hexa- (17.2%), penta- (10.7%), tetra- (4.4%), and higher-order repeats (1.6%)(Table 1). Almost half of these unigenes were not suitable for marker development, because their SSR-flanking sequences were too short for designing PCR primers. Consequently, a total of 4,713 primer pairs were successfully designed, and 1,141 of them were selected for further analysis.

**Table 1:** Occurrence of SSRs in the transcriptome of tea plant.

Repeat type	Number	Proportion (%)	Frequency (%)	Average distance (kb/SSR)
Dinucleotide	3,595	38.9	6.0	10.23
Trinucleotide	2,514	27.2	4.2	14.62
Tetranucleotide	408	4.4	0.7	90.14
Pentanucleotide	989	10.7	1.7	37.18
Hexanucleotide	1,586	17.2	2.7	23.18
Higher-order	147	1.6	0.3	250.19
Total	9,239	100	15.6	3.98

### Marker polymorphism

Out of the 1,509 genic and genomic SSR primers screened, 450 (29.8%) exhibited reproducible amplification and distinct polymorphism between the mapping parents (Table 2). The level of polymorphism was significantly higher for genic SSR markers (30.6%) compared with genomic SSR markers (6.1%). The female parent ‘YS’ was less heterozygous than the male parent ‘BD’. Of the 450 informative marker loci, 198 segregated in both parents (44.0%): 13 with four segregating alleles, 127 with three alleles, 58 with two alleles; and in parallel, 74 segregated only in ‘YS’ (16.4%) while 178 only in ‘BD’ (39.6%). Primer sequences and characteristics of the newly developed genic SSR markers are provided in Table S1.

**Table 2:** Summary of marker sets and the informative SSR markers validated in the ‘YS’×‘BD’ tea plant population.

Type	Marker set	Acronym	Tested	Mappable	Segregation type				
					lm×ll	nn×np	hk×hk	ef×eg	ab×cd
Genic SSR	Newly developed^a^	TM	1,141	328	62	135	33	88	10
	Taniguchi et al. [25]	MSE/MSG	45	26	4	10	0	10	2
	Jin et al. [34]	P	10	4	0	2	0	2	0
	Sharma et al. [36]	TUGMS	61	15	2	6	1	5	1
	Ma et al. [37], [40]	TM	104	37	2	9	13	13	0
	Zhou et al. [38]	TM	59	25	4	10	6	5	0
	Yao et al. [41]	TM	40	12	0	5	5	2	0
Genomic SSR	Freeman et al. [29]	CamsinM	13	1	0	0	0	1	0
	Hung et al. [30]	Ca	11	0	0	0	0	0	0
	Chen et al. [31] b	CN	10	1	0	0	0	1	0
	Yang et al. [32] c	A	15	1	0	1	0	0	0
Total			1,509	450	74	178	58	127	13

a Novel genic SSRs developed from the transcriptome of *C. sinensis*.

b,c Genomic SSRs derived from *C.nitidissima* Chi and *C. taliensis* (W. W. Sm.) Melch, respectively.

### Segregation of the markers

For each of the 450 informative SSR markers, genotypes were obtained for all 183 offspring. Chi-square analysis of genotypic data for the mapping population revealed that 320 (71.1%) loci fitted the expected Mendelian ratio, while 42 (9.3%) loci exhibited slight SD (0.01<*P*≤0.05) and 88 (19.6%) were severely distorted (*P*≤0.01). Among the 130 skewed marker loci, 68 (52.3%) showed distortion in favor of the ‘YS’ genotype; 15 (11.5%) showed distortion in the direction of the ‘BD’ genotype; 11 (8.5%) were deviated towards the parental genotypes; and 36 (27.7%) were skewed towards the heterozygous genotypes (Table 3).

**Table 3:** Summary of skewed SSR markers.

Segregation type	Number of distorted markers				
	I*	II*	III*	IV*	Total
ab×cd	-	-	-	2	2
ef×eg	16	4	3	19	42
hk×hk	-	-	8	15	23
lm×ll	11	1	-	-	12
nn×np	41	10	-	-	51
Total	68	15	11	36	130

* Markers exhibiting skewed genotypic frequencies toward maternal parent ‘YS’ (I), paternal parent ‘BD’ (II), both parents (III) and heterozygotes (IV), respectively.

### Linkage map

**Figure 1:**
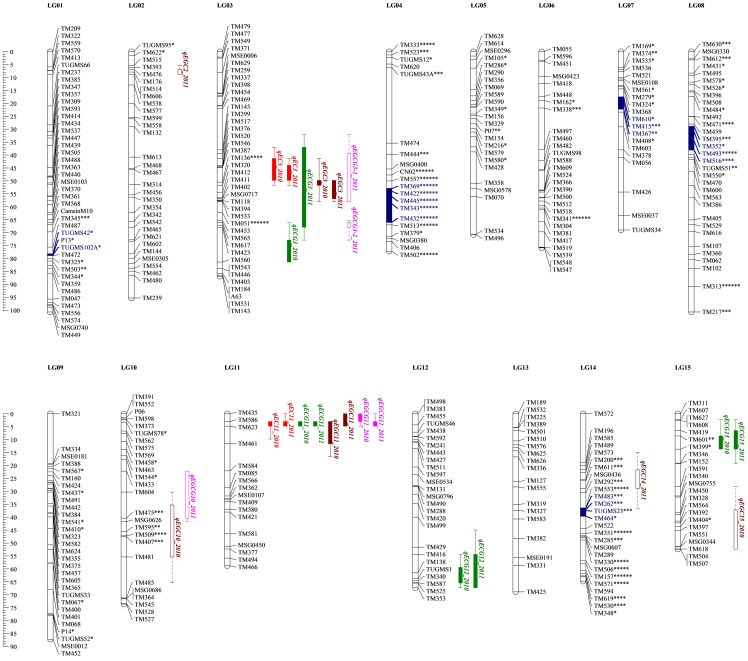
SSR-based genetic map of tea plant showing location of QTLs for catechins content identified in the ‘YS’×‘BD’ mapping population. Map distance scales in cM are placed at left margin. Markers with distorted segregation ratios are marked with *asterisks* according to their significance levels (*: 0.05, **: 0.01, ***: 0.005, ****: 0.001, *****: 0.0005, ******: 0.0001). Regions of linkage groups considered to be candidate segregation distortion region (SDR) are denoted by *blue fill*. Significant QTL for each catechin is represented by different color (EC, red; ECG, green; EGC, coffee; EGCG, pink). *Bars and lines* indicate 1-LOD and 2-LOD support intervals. The *solid bars and lines* indicate stable QTL (detected across years), and *empty bars and dashed* lines putative QTL (only detected in one year). *EC* epicatechin, *ECG* epicatechin gallate, *EGC* epigallocatechin, *EGCG* epigallocatechin gallate.

Genotypic data of all 450 loci were used for linkage analysis. As a result, 406 SSR markers were finally mapped into 15 linkage groups, corresponding to the haploid chromosome number of tea plant (Table 4; Figure 1). The linkage map covered a total genetic distance of 1,143.5 cM, with an average locus spacing of 2.9 cM. Individually, the linkage groups ranged in size from 52.4 cM (LG15) to 100.7 cM (LG01 and LG08), and the number of markers on each group varied from 17 for LG11 to 43 for LG01 (Table 4). LG03 possessed the highest marker density with an average locus distance of 2.2 cM, while the other LGs had relatively lower marker density (2.3–3.9 cM/SSR). The majority of spaces between two adjacent markers were shorter than 20 cM, and there was only one larger marker interval, distributing on LG04 (TUGMS43A-TM474) with a map distance of 29.3 cM.

**Table 4:** Marker distribution among linkage groups.

Linkage group	Number of markers	Length (cM)	Average distance between adjacent markers (cM)	Number of anchor markers^a^	Length of linkage group in reference map (cM)^b^	Percentage covering of reference map^c^	No. of distorted markers
LG01	43	100.7	2.3	2	110	92	7
LG02	31	95.3	3.1	1	104	92	2
LG03	40	86.9	2.2	2	97	90	2
LG04	20	77.2	3.9	2	98	79	15
LG05	24	70.8	3.0	2	67	106	6
LG06	28	75.9	2.7	1	86	88	3
LG07	20	69.0	3.5	2	84	82	10
LG08	32	100.7	3.1	1	90	112	15
LG09	30	81.2	2.7	2	85	96	7
LG10	26	73.6	2.8	2	78	94	7
LG11	17	58.8	3.5	2	74	79	0
LG12	26	67.3	2.6	2	60	112	0
LG13	19	68.9	3.6	1	62	111	0
LG14	27	64.8	2.4	2	62	105	17
LG15	23	52.4	2.3	2	61	86	3
Total/average	406	1143.5	2.9	26	1218	94	94

a SSR markers mapped on both ‘YS’×‘BD’ linkage map and reference map.

b Reference map was constructed based on the ‘Sayamakaori’×‘Kana-Ck17’ mapping population [25].

c Length of ‘YS’×‘BD’ linkage map expressed as a percentage of length of reference linkage map.

Based on the anchor markers which were mapped both in the current map and the reference map (published by Taniguchi et al. [25]), the relationships between LGs of two maps were established (Figure S2). Each LG contained at least one anchor locus, and the map position of these loci showed good collinearity between the homologous LGs. The newly developed genetic map covered 94% of 1,212 cM of the reference map, and the lengths of homologous LGs of two maps were generally concordant (Table 4).

The linkage map contained a total of 94 distorted markers which were marked with *asterisks* and denoted on the map according to their distortion level (Figure 1). The majority of these markers (68.1%) were clustered on five linkage groups (LG01, LG04, LG07, LG08, and LG14), and the remaining 31.9% were randomly distributed on other linkage groups except for LG11, LG12, and LG13. Fifteen (75%) mapped markers on LG04 were severely distorted (*P*<0.001) and it was 17 (63%) for LG14.

To test whether the genotypic SD was caused by gametic and/or zygotic selection, segregation patterns of skewed markers were analyzed using the method as described by Li et al. [56]. In brief, gametic selection (allelic SD test) was demonstrated by testing the deviation of the observed allelic distribution from the expected Mendelian ratio for each heterozygous parent, and zygotic selection (zygotic SD test) was assessed by testing the observed genotypic distribution from the genotypic ratio that was expected given allele frequency estimates (for details see Li et al. [56]). Any region with at least three adjacent marker loci showing significant SD was considered as a candidate segregation distortion region (SDR), wherein the skewed markers showed similar SD patterns and had similar gametic and zygotic selection tests.

Out of 94 total skewed markers, only six showed significant zygotic SD (*P*<0.05) while 85 (90.4%) were significantly distorted based on combination of parental allelic SD tests (Table S2). Thus gametic selection seemed to play a major role in contributing to the SD in the present mapping population. Five candidate SDRs were identified on five LGs (*blue-filled* LG segments in Figure 1). The LG04 SDR contained five distorted makers, covering a map distance of 13.0 cM; and the SDRs on LG08 and LG14 both comprised four SD loci, while it was three respectively for LG01 and LG07. All the markers within five SDRs showed allelic SD but not zygotic SD. Of these, the markers on LG14 exhibited excess heterozygosity, while the markers on other four LGs exhibited skewed genotypic frequencies toward maternal parent ‘YS’ (Table S2).

### Catechins content

The principal components of tea catechins, including EC, ECG, EGC, and EGCG, were quantified in F_1_ progeny and both parents in two measurement years. Mean value, standard deviation, range, and coefficient of variation for each catechin are shown in Table 5. Overall, EGCG was the most abundant of catechin compounds. Significant differences were observed between the parents for all catechins, with the exception of EGCG in 2010. The male parent ‘BD’ had higher content of EC and ECG than the female parent ‘YS’, while the content of EGC and EGCG varied considerably between years.

**Table 5:** Phenotypic variation of individual catechins content in parental lines (‘YS’ and ‘BD’) and 183 F_1_ progeny.

Trait	Year	YS	BD	F_1_		
		Mean ± SD	Mean ± SD	Mean ± SD	Range	CV (%)
EC (mg/g)	2010	9.46±0.09	19.33±0.11	12.50±3.61	6.55–29.72	28.9
	2011	6.37±0.56	18.08±0.36	11.90±3.78	5.26–24.59	31.8
ECG (mg/g)	2010	24.38±0.38	61.24±1.33	29.20±8.87	12.28–57.04	30.4
	2011	23.56±0.45	58.63±1.54	31.90±9.80	17.64–65.81	30.7
EGC (mg/g)	2010	36.72±0.60	34.61±1.06	34.79±11.95	7.91–69.68	34.3
	2011	17.94±0.65	33.37±1.82	23.68±7.65	8.02–44.42	32.3
EGCG (mg/g)	2010	160.96±2.59	139.88±2.71	124.86±15.09	90.17–159.58	12.1
	2011	154.32±2.71	156.51±4.30	118.41±15.30	86.48–157.37	12.9

*EC* epicatechin, *ECG* epicatechin gallate, *EGC* epigallocatechin, *EGCG* epigallocatechin gallate, *SD* standard deviation, *CV* coefficient of variation.

High levels of variation in catechins content were observed in F_1_ progeny (Table 5). The means of the population were towards the lower end of ranges for all catechin compounds, and each of them exhibited a continuous variation with transgressive segregation, indicating a polygenic inheritance (Figure S3). Pearson's correlations between catechin compounds were calculated in Table 6. The data show that the individual catechins correlated significantly with each other, with the strongest positive correlation between EC and ECG (*r* = 0.61, *P*<0.001), and the strongest negative correlation between ECG and EGCG (*r* = −0.52, *P*<0.001).

**Table 6:** Pearson's correlations between the individual catechin compounds.

	EC	ECG	EGC
ECG	0.61***		
EGC	0.36***	−0.40***	
EGCG	−0.47***	−0.52***	0.23**

*EC* epicatechin, *ECG* epicatechin gallate, *EGC* epigallocatechin, *EGCG* epigallocatechin gallate.

**P<0.01,

***P<0.001.

### Identified QTLs

A total of 25 QTLs associated with catechins content of tea plant were detected over two measurement years by both IM and rMQM analyses at the significant genome-wide threshold of 5% (Table S3). Of these, nine stable QTLs were validated across years (Table 7), and mainly clustered into four genomic regions on LG03, LG11, LG12 and LG15 (Figure 1). The other QTLs, only detected in one year and therefore considered as putative QTLs, were located on LG02, LG03, LG10, LG14 and LG15. The population variability explained by each QTL was predominantly at moderate-to-high levels and ranged from 2.4% to 71.0%, with an average of 17.7%. Thirteen major QTLs were identified for four traits in three main regions on LG02, LG03 and LG11 (Table S3). The total number of QTL for each trait varied from four (EC) to eight (ECG and EGC), while the total population variability explained by all QTLs for a trait ranged between 38.4% (EC 2011) and 79.7% (ECG 2011). For most of QTLs, the additive effects from maternal parent ‘YS’ (a1) were significant higher than those from paternal parent ‘BD’ (a2), indicating that the alleles for increasing catechins content at these loci were probably mainly contributed by the parents of ‘YS’.

**Table 7:** Overview of the stable (detected across years) QTLs for catechins content detected in the ‘YS’×‘BD’ tea plant population.

Trait	QTL	LG	Year	Positon (cM ± 2-LOD)	LOD threshold^a^	LOD score	PVE (%)	Nearest marker	a1^b^	a2^b^	d^c^
EC (mg/g)	*qEC3*	3	2010	44.0 (37.0–51.7)	4.2	6.39	10.5	TM376	4.34	0.63	1.68
			2011	44.7 (41.3–51.7)	4.3	7.88	14.5	TM546	4.97	2.54	1.65
	*qEC11*	11	2010	4.8 (3.0–9.8)	4.2	14.86	31.2	TM623	7.28	−4.33	−2.64
			2011	3.0 (0–4.8)	4.3	10.52	23.9	TM586	7.15	−1.89	−2.48
ECG (mg/g)	*qECG3*	3	2010	77.9 (66.2–81.4)	4.2	5.09	2.6	TM560	−3.14	5.55	0.34
			2011	65.4 (32.0–72.9)	4.2	7.2	3.9	TM453	−3.42	5.87	2.46
	*qECG11*	11	2010	3.0 (3.0–4.8)	4.2	52.85	71.0	TM586	29.68	−10.79	−6.35
			2011	3.0 (3.0–4.8)	4.2	55.63	69.7	TM586	33.29	−8.32	−6.62
	*qECG12*	12	2010	64.6 (54.4–67.3)	4.2	4.66	2.5	TM340	4.71	0.80	2.53
			2011	59.4 (45.0–67.3)	4.2	4.81	2.4	TM138	3.88	2.71	3.65
	*qECG15*	15	2010	13.6 (2.3–13.7)	4.2	7.17	3.5	TM399	−1.30	−7.09	2.38
			2011	13.6 (2.3–19.2)	4.2	7.93	3.7	TM399	0.54	−7.94	1.03
EGC (mg/g)	*qEGC3*	3	2010	50.5 (41.3–58.0)	4.3	7.25	11.9	TM136	16.72	1.18	1.80
			2011	54.4 (49.8–58.0)	4.2	9.26	14.9	TM412	11.45	4.77	2.03
	*qEGC11*	11	2010	4.8 (3.0–16.6)	4.3	10.54	19.3	TM623	−20.30	−2.25	−2.89
			2011	0 (0–4.8)	4.2	7.21	12.3	TM435	−10.79	1.56	3.27
EGCG (mg/g)	*qEGCG11*	11	2010	0 (0–4.8)	4.3	17.9	40.1	TM435	−38.59	2.77	3.26
			2011	0 (0–4.8)	4.3	29.13	52.4	TM586	−42.67	3.78	3.64

*EC* epicatechin, *ECG* epicatechin gallate, *EGC* epigallocatechin, *EGCG* epigallocatechin gallate, *LOD* logarithm of odds ratio, *PVE* phenotypic variation explained.

a The genome-wide LOD significance thresholds (*P*<0.05) based on permutation testing (n = 1000).

b a1and a2 represent the additive (or average allele substitution) effects from maternal parent and paternal parent, respectively.

c The overall dominance effects.

#### QTL for EC content

Two major and stable QTLs, *qEC3* and *qEC11*, were identified for EC content, accounting for a total of 41.7% and 38.4% of the population variability in 2010 and 2011 respectively (Figure 1; Table 7). The *qEC3* was located on LG03 close to markers TM376-TM546 with an average PVE of 12.5% over the years, while *qEC11* located on LG11 close to markers TM623-TM586 with an average of 27.6% of the population variability. The two QTLs both showed positive additive effect a1, indicating that the allele leading to an increase for EC was contributed by ‘FD’ (the female parent of ‘YS’). The average increased effect was 4.66 and 7.21 mg respectively for *qEC3* and *qEC11*.

#### QTL for ECG content

Four stable QTLs were detected for ECG content, collectively explained 79.6% and 79.7% of the total population variability in 2010 and 2011 respectively (Figure 1; Table 7). QTL distributed across four LGs, with a LOD score ranging from 4.66 to 55.63. The major QTL, qECG11, was located on LG11 close to marker TM586 and had the largest effect on ECG content, accounting for an average of 70.4% of the population variability over the years. The allele contributed to additive effect a1 at this locus came from ‘FD’ and increased ECG by 31.48 mg, on average over the years. The other three QTLs with small effects were detected on LG03, LG12 and LG15. These QTL (qECG3, qECG12 and qECG15) explained an average of 3.3, 2.5 and 3.6% of the population variability, respectively.

#### QTL for EGC content

A total of eight QTLs were detected for EGC content, accounting for a total of 43.5% and 49.4% of the population variability in 2010 and 2011 respectively (Figure 1; Table S3). Of these, two major and stable QTLs, *qEGC3* and *qEGC11*, were mapped on LG03 and LG11, respectively (Table 7). The *qEGC3* was located close to marker TM136-TM412 with an average PVE of 13.4% over the years, while the *qEGC11* was located close to marker TM623-TM435 with an average PVE of 15.8%. The *qEGC3* showed a positive a1 meaning that the allele for increasing EGC was from ‘FD’, whereas *qEGC11* showed a negative a1 indicating allele for EGC in ‘YD’. The increased effect was 14.09 and 15.55 mg respectively for *qEGC3* and *qEGC11*. An additional four putative QTLs were identified on LG02, LG10, LG14 and LG15 (Table S3). The LOD score for these loci varied from 4.25 to 9.68, and the PVE ranged between 5.0% and 15.7%.

#### QTL for EGCG content

One major and stable QTL, *qEGCG11*, was identified for EGCG content on LG11, close to marker TM435-TM586, contributing to 46.3% of the population variability on average over the years (Figure 1; Table 7). The additive effect a1 of the ‘YD’ allele at this locus increased EGCG on average by 40.63 mg. In addition, three putative QTLs was mapped on LG03 and LG10, and explained between 2.4 and 8.6% of the population variability (Table S3).

## Discussion

### Genic SSRs derived from the tea plant transcriptome

In the past several years, large-scale sequencing and the application of next-generation sequencing (NGS) technologies have exponentially increased the volume of *in silico* databases of nucleotide sequences, from which SSR markers can be rapidly developed at low cost [57], [58]. Although SSR mining is not usually the first priority of such sequencing projects, the enormous amounts of sequence data can be used for this purpose [59]. In this case, a total of 9,239 genic SSRs were identified based on the 59,962 non-redundant unigene sequences generated from the transcriptome assembly in our previous study [44]. About 12.7% of the unigenes possess SSR loci. The average frequency of SSRs was about 1/3.98 kb, which is higher than that estimated by Wu et al. (1/4.99 kb) using a set of 25,637 tea plant unigenes [43]. This is probably because the search parameters used for SSR exploration, e.g. the number of repeat motifs unit, were quite different between the two studies. Dinucleotide repeat motifs were the predominant repeat type among the unigenes analyzed herein, accounting for 38.9% of the total SSR loci identified, followed by trinucleotide (27.2%), hexanucleotide (17.2%), pentanucleotide (10.7%), tetranucleotide (4.4%), and higher-orders (1.6%). These results are generally consistent with the findings of Taniguchi et al. [42] and Wu et al. [43]. Out of the 1141 newly designed SSR primer pairs, 831 (73%) could yield amplicons in the mapping parents, comprising of 361 (32%) monomorphic and 470 (41%) polymorphic marker loci (Table S4). The polymorphic ratio is similar to those obtained in previous studies in tea plant (ranging from 31% to 70%) [25], [36], [39], [40], [42], [43]. Among the polymorphic SSRs, 328 loci were heterozygous in at least one parent with either two or three alleles, indicating that these loci can be used for linkage analysis in the present mapping population. The proportion of mappable SSR markers is comparable with that reported by Taniguchi et al. [25]. Overall, deep transcriptome sequencing in tea plant offers an excellent opportunity to quickly identify a large number of genic SSRs.

### Genetic map of tea plant

A saturated genetic map can be a valuable tool for genetic research and molecular breeding. The first genetic map in tea plant was reported by Tanaka et al. [19] in a ‘Yabukita’×‘Shizuka-Inzatsu 131’ pseudo-testcross population with randomly amplified polymorphic DNA (RAPD) markers. But that map contained only six linkage groups and very small numbers of markers. Later, several genetic maps of tea plant were constructed based on dominant marker systems, including RAPD, inter-simple sequence repeats (ISSR), and amplified fragment length polymorphisms (AFLP), with a total coverage from 1,180 to 2,545 cM [20]–[24]. However, the numbers of mapped markers on these linkage maps were still relatively small, ranging from 62 to 208. Recently, a high-density reference linkage map has been developed based on a ‘Sayamakaori’×‘Kana-CK17’ tea plant population [25]. The combined map contain 441 SSRs, 7 CAPS, 2 STS and 674 RAPDs, and the numbers of linkage groups are coincided with the basic number of chromosomes in tea plant (n = 15). But the mapping population used in that case is relatively small, and there are several large gaps between adjacent markers in some linkage groups which may decrease the power of QTL detection and affect the accuracy of QTL effect estimation for QTLs inside or near the gaps. Therefore, more genetic maps need to be constructed using much bigger mapping populations.

In this study, we developed a moderately saturated genetic map using SSR markers, based on a population of 183 individuals derived from the cross of two *C. sinensis* varieties. The total map length was 1,143.5 cM with 406 markers, which is close to the estimates for tea plant genome reported by Ota and Tanaka [20] (1640 cM), Hackett et al. [21] (1349.7 cM), and Taniguchi et al. [25] (1,298 and 1,305 cM), but considerably smaller than those obtained by Huang et al. [22] (2,457.7 and 2,545.3 cM), and Hu et al. [26] (4,482.9 cM). Although an increase in total map length would generally mean more genome coverage, a direct comparison of these maps was not possible. Because the accuracy of the genetic distance estimates was determined by several factors, including size and type of mapping populations, number and type of mapped marker loci, mapping strategies, statistical algorithm and computer packages [60]–[64].

The average interval between two markers was 2.9 cM in the current genetic map, which is comparable with the marker densities for historic maps (ranging from 1.9 to 19.0 cM) [19]–[26]. In addition, the mapped markers were generally uniform distributed, and all linkage groups showed similar marker density, ranging from 2.2 to 3.9 cM (Table 4). The recommended marker density for genome-wide QTL mapping is a mean inter-marker interval of less than 10 cM [65], [66]. Thus the genetic map constructed in the present study is suitable for QTL identification. However, there was still one gap of larger than 20 cM between adjacent markers on LG04. The presence of large gaps may be explained in three ways. Firstly, the genic SSR markers were derived from the transcriptome sequences, indicating that the genic marker based linkage maps represent only expressed regions of the genome (mostly the euchromatic regions), and thus heterochromatin and other repeat regions may be underrepresented, leading to large physical gaps between genic markers spanning these regions [66]. Secondly, the genome regions, corresponding to the gap regions of genetic map, are homozygous in both mapping parents; hence no recombination can be detected in this case. Thirdly, the individuals in the mapping population were not enough to observe the recombination in the gap regions. Presence of large gaps in the genetic map may lead to failure in detection of QTLs in these regions. Thus further studies will be needed to fill this gap in the present genetic map.

In order to establish the relationships with the reference map [25], we selected 45 anchor markers from the reference map for linkage mapping. Finally, 26 of these markers were mapped into the present genetic map, distributing on 15 linkage groups (Figure 1). According to these anchor loci, the homologous LGs of two maps were validated, and showed a good collinearity (Figure S2). With a total genetic distance of 1,143.5 cM, the present map is almost the same length as the reference map (1,212 cM), indicating a considerable level of genome coverage. In consequence this new genic linkage map will provide a foundation on which a wide variety of genomic and genetic research can be built, facilitating molecular breeding of tea plant.

### Segregation distortion

Segregation distortion is a phenomenon that genotypic frequencies of a locus deviate from the expected Mendelian ratios [67] and has been described in many species, such as maize [68], wheat [69], mungbean [70], *Eucalyptus* [71] and *Populus* [72]. In tea plant, SD is a feature of most mapping populations [21]–[24], [26]. In this case, a total of 30.7 percent of the tested markers were significant distorted (*P*<0.05). This rate is in the range of those previously obtained in tea plant (ranging from 12.0–32.9%) [21]–[24], [26]. It has already been proven that the markers with significant SD have slight impact on map order or length [73], [74]; hence the distorted markers were usually discarded in subsequent linkage analyses. However, in order to better understand the reason for SD in the current mapping population, we still used the skewed markers for linkage mapping, with the exception of those greatly affecting marker orders or excessively changing linkage distances. Finally, 94 of the skewed markers were mapped onto the current genetic map, distributing in twelve linkage groups.

There are several factors that contribute to SD, including non-biological factors (sample size and genotyping errors), and biological factors (gametic and/or zygotic selection) [69]. The percentage of markers with SD caused by the former factors is variable, while SD caused by the latter are generally believed to be related to genes that are subject to direct (gametic) selection [75]. In this case, the results of allelic and zygotic tests showed that most of the mapped skewed markers displayed allelic SD but not zygotic SD, suggesting that gametic selection may be the underlying reason for genotypic SD in the present mapping population. And the five candidate SDRs also suggest that their corresponding regions in the tea plant genome may contain genes that affect viability or fitness. However, a more in-depth study may be required to further investigate this possibility.

### QTLs for catechins content

The dissection of QTL for important agronomic traits and validation of marker-trait associations can facilitate selection of plants with desired features at early stages of growth. This is particularly valuable for woody plants such as tea plant, because the breeding program based on conventional phenotypic selection in these plants is often delayed due to their long juvenile stage. Catechins are one of the most important chemical components of tea leaves, and can greatly affect the tea quality. However, the genetic basis of this phenotype remains poorly understood. In the present study, we firstly reported the mapping of QTLs for catechins content in tea plant. In total, 25 tea catechins QTLs were detected, and the population variability explained by each QTL varied from 2.4% to 71.0%, with an average of 17.7%. The high level of PVE exhibited by these QTLs suggests that the catechins content may be controlled by only a few critical genes. However, the relative small size of mapping population used herein may lead to the overestimation of QTL effects and the decrease of statistical power for detection of QTLs with smaller effects [76]. Thus for further investigation, we need an increase in the size of our mapping population for a better estimation of QTL location and effect.

The accuracy of locating QTL is also strongly influenced by environmental effects, because some environment-specific QTLs may express differently in different environments [76], [77]. These QTLs can be difficult or impossible to use in breeding for improvement of functional traits. In this case, seven QTLs were detected to be significant in only one growing year, while nine stable QTLs were validated across two years. And among these stable QTLs, six had major effect on catechins content, including at least one major QTL per trait. More interestingly, the major QTLs detected showed a marked tendency to co-localize, clustering on LG03 and LG11. It is particularly prominent in the region between 0 and 16.6 cM of LG11, where four major QTLs are located, one per trait. This chromosomal region probably contains multifunctional genes associated with catechins production and accumulation, and deserves further investigation.

## Supporting Information

Figure S1 **HPLC chromatograms of (A) catechin standard mixture, (B) tea catechins extracted from a typical tea sample (THB-010).**
(TIF)

Figure S2 **Comparison between the homologous linkage groups (LGs) of the ‘YS’×‘BD’ genetic map and the reference map of tea plant.** The reference map is listed on the right in each LG, representing as LG01_Core to LG15_Core (published by Taniguchi et al. [25]). Map distance scales in Kosambi centimorgans (cM) are placed at left margin. *Lines* connect anchor markers.
(TIF)

Figure S3 **Frequency distribution patterns of catechins content in F_1_ population derived from the cross between ‘YS’ and ‘BD’.** Parental values are indicated with *arrows*.
(TIF)

Table S1 **Primer sequences and characteristics of the novel genic SSR markers developed from the transcriptome of tea plant.**
(PDF)

Table S2 **Marker names, linkage groups, segregation types, segregation distortion (SD) patterns, and p values for tests of genotypic segregation distortion, for allelic SD in each parent, and for zygotic SD in the ‘YS’×‘BD’ population.**
(PDF)

Table S3 **Summary of the significant QTLs (detected in two measurement years) evidenced by both single-QTL model (IM) and restricted multiple QTL model (rMQM) mapping for catechins content using the ‘YS’×‘BD’ tea plant population.**
(PDF)

Table S4 **Summary of primer screening of the novel genic SSRs based on PCR amplification.** DNA from the mapping parents was used to verify the polymorphisms of the SSR primers.
(PDF)

## References

[1] International Tea Committee (2012) Annual bulletin of statistics 2012. London: ITC. pp. 50–60.

[2] Zaveri NT (2006) Green tea and its polyphenolic catechins: medicinal uses in cancer and noncancer applications. Life Sci 78: 2073–2080. 16445946 10.1016/j.lfs.2005.12.006

[3] Basu A, Lucas EA (2007) Mechanisms and effects of green tea on cardiovascular health. Nutr Rev 65: 361–375. 17867370 10.1301/nr.2007.aug.361-375

[4] Weinreb O, Amit T, Mandel S, Youdim MB (2009) Neuroprotective molecular mechanisms of (-)-epigallocatechin-3-gallate: a reflective outcome of its antioxidant, iron chelating and neuritogenic properties. Genes Nutr 4: 283–296. 19756809 10.1007/s12263-009-0143-4 PMC2775893

[5] Yang CS, Wang X, Lu G, Picinich SC (2009) Cancer prevention by tea: animal studies, molecular mechanisms and human relevance. Nat Rev Cancer 9: 429–439. 19472429 10.1038/nrc2641 PMC2829848

[6] Wang H, Provan GJ, Helliwell K (2000) Tea flavonoids: their functions, utilisation and analysis. Trends Food Sci Tech 11: 152–160.

[7] Vuong QV, Stathopoulos CE, Nguyen MH, Golding JB, Roach PD (2011) Isolation of green tea catechins and their utilization in the food industry. Food Rev Int 27: 227–247.

[8] Pang Y, Abeysinghe IS, He J, He X, Huhman D, et al (2013) Functional characterization of proanthocyanidin pathway enzymes from tea and their application for metabolic engineering. Plant Physiol 161: 1103–1116. 23288883 10.1104/pp.112.212050 PMC3585583

[9] Saravanan M, Maria John KM, Raj Kumar R, Pius PK, Sasikumar R (2005) Genetic diversity of UPASI tea clones (*Camellia sinensis* (L.) O. Kuntze) on the basis of total catechins and their fractions. Phytochemistry 66: 561–565. 15721948 10.1016/j.phytochem.2004.06.024

[10] Schramm L (2013) Going Green: The role of the green tea component EGCG in chemoprevention. J Carcinog Mutagen 4: 1000142. 24077764 10.4172/2157-2518.1000142 PMC3783360

[11] Tounektia T, Joubertbc E, Hernándezd I, Munné-Boschd S (2013) Improving the polyphenol content of tea. Crit Rev Plant Sci 32: 192–215.

[12] Tanaka J, Taniguchi F, Hirai N, Yamaguchi S (2006) Estimation of the genome size of tea (*Camellia sinensis*), camellia (*C. japonica*), and their interspecific hybrids by flow cytometry. Tea Res J 101: 1–7.

[13] Collard BC, Mackill DJ (2008) Marker-assisted selection: an approach for precision plant breeding in the twenty-first century. Phil Trans R Soc Lond B 363: 557–572. 17715053 10.1098/rstb.2007.2170 PMC2610170

[14] Xu Y, Crouch JH (2008) Marker-assisted selection in plant breeding: from publications to practice. Crop Sci 48: 391–407.

[15] Miura K, Ashikari M, Matsuoka M (2011) The role of QTLs in the breeding of high-yielding rice. Trends Plant Sci 16: 319–326. 21429786 10.1016/j.tplants.2011.02.009

[16] Wallace JG, Larsson SJ, Buckler ES (2013) Entering the second century of maize quantitative genetics. Heredity 112: 30–38. 23462502 10.1038/hdy.2013.6 PMC3860165

[17] Foolad MR, Panthee DR (2012) Marker-assisted selection in tomato breeding. Crit Rev Plant Sci 31: 93–123.

[18] Barchi L, Lanteri S, Portis E, Valè G, Volante A, et al (2012) A RAD tag derived marker based eggplant linkage map and the location of QTLs determining anthocyanin pigmentation. PLoS ONE 7: e43740. 22912903 10.1371/journal.pone.0043740 PMC3422253

[19] Tanaka J, Sawai Y, Yamaguchi S (1995) Genetic analysis of RAPD markers in tea. Jpn J Breed 45: 198.

[20] Ota S, Tanaka J (1999) RAPD-based linkage mapping using F_1_ segregating populations derived from crossings between tea cultivar ‘Sayamakaori’ and Strain ‘Kana-Ck17’. Breed Res 1: 16.

[21] Hackett CA, Wachira FN, Paul S, Powell W, Waugh R (2000) Construction of a genetic linkage map for *Camellia sinensis* (tea). Heredity 85: 346–355. 11122412 10.1046/j.1365-2540.2000.00769.x

[22] Huang JA, Li JX, Huang YH, Luo JW, Gong ZH, et al (2005) Construction of AFLP molecular markers linkage map in tea plant. J Tea Sci 25: 7–15.

[23] Huang FP, Liang YR, Lu JL, Chen RB (2006) Genetic mapping of first generation of backcross in tea by RAPD and ISSR markers. J Tea Sci 26: 171–176.

[24] Kamunya SM, Wachira FN, Pathak RS, Korir R, Sharma V, et al (2010) Genomic mapping and testing for quantitative trait loci in tea (*Camellia sinensis* (L.) O. Kuntze). Tree Genet Genomes 6: 915–929.

[25] Taniguchi F, Furukawa K, Ota-Metoku S, Yamaguchi N, Ujihara T, et al (2012) Construction of a high-density reference linkage map of tea (*Camellia sinensis*). Breed Sci 62: 263–273. 23226087 10.1270/jsbbs.62.263 PMC3501944

[26] Hu CY, Lee TC, Tsai HT, Tsai YZ, Lin SF (2013) Construction of an integrated genetic map based on maternal and paternal lineages of tea (*Camellia sinensis*). Euphytica 191: 141–152.

[27] Varshney RK, Graner A, Sorrells ME (2005) Genic microsatellite markers in plants: features and applications. Trends Biotechnol 23: 48–55. 15629858 10.1016/j.tibtech.2004.11.005

[28] Gupta PK, Varshney RK (2000) The development and use of microsatellite markers for genetic analysis and plant breeding with emphasis on bread wheat. Euphytica 113: 163–185.

[29] Freeman S, West J, James C, Lea V, Mayes S (2004) Isolation and characterization of highly polymorphic microsatellites in tea (*Camellia sinensis*). Mol Ecol Notes 4: 324–326.

[30] Hung CY, Wang KH, Huang CC, Gong X, Ge XJ, et al (2008) Isolation and characterization of 11 microsatellite loci from *Camellia sinensis* in Taiwan using PCR-based isolation of microsatellite arrays (PIMA). Conserv Genet 9: 779–781.

[31] Chen ZY, Jiang YS, Wang ZF, Wei JQ, Wei X, et al (2000) Development and characterization of microsatellite markers for *Camellia nitidissima* . Conserv Genet 11: 1163–1165.

[32] Yang JB, Yang J, Li HT, Zhao Y, Yang SX (2009) Isolation and characterization of 15 microsatellite markers from wild tea plant (*Camellia taliensis*) using FIASCO method. Conserv Genet 10: 1621–1623.

[33] Bali S, Raina SN, Bhat V, Aggarwal RK, Goel S (2013) Development of a set of genomic microsatellite markers in tea (*Camellia* L.) (Camelliaceae). Mol Breeding 32: 735–741.

[34] Jin JQ, Cui HR, Chen WY, Lu MZ, Yao YL, et al (2006) Data mining for SSRs in ESTs and development of EST-SSR marker in tea plant (*Camellia sinensis*). J Tea Sci 26: 17–23.

[35] Zhao LP, Liu Z, Chen L, Yao MZ, Wang XC (2008) Generation and characterization of 24 novel EST derived microsatellites from tea plant (*Camellia sinensis*) and cross-species amplification in its closely related species and varieties. Conserv Genet 9: 1327–1331.

[36] Sharma RK, Bhardwaj P, Negi R, Mohapatra T, Ahuja PS (2009) Identification, characterization and utilization of unigene derived microsatellite markers in tea (*Camellia sinensis* L.). BMC Plant Biol 9: 53. 19426565 10.1186/1471-2229-9-53 PMC2693106

[37] Ma JQ, Zhou YH, Ma CL, Yao MZ, Jin JQ (2010) Identification and characterization of 74 novel polymorphic EST-SSR markers in the tea plant, *Camellia sinensis* (Theaceae). Am J Bot 97: e153–156. 21616837 10.3732/ajb.1000376

[38] Zhou YH, Qiao XY, Ma CL, Qiao TT, Jin JQ, et al (2011) Genetic diversity and structure of tea landraces from guangxi based on EST-SSR analysis. Sci Silvae Sin 47: 59–67.

[39] Sharma H, Kumar R, Sharma V, Kumar V, Bhardwaj P (2011) Identification and cross-species transferability of 112 novel unigene-derived microsatellite markers in tea (*Camellia sinensis*). Am J Bot 98: e133–138. 21653500 10.3732/ajb.1000525

[40] Ma JQ, Ma CL, Yao MZ, Jin JQ, Wang ZL, et al (2012) Microsatellite markers from tea plant expressed sequence tags (ESTs) and their applicability for cross-species/genera amplification and genetic mapping. Sci Hortic 134: 167–175.

[41] Yao MZ, Ma CL, Qiao TT, Jin JQ, Chen L (2012) Diversity distribution and population structure of tea germplasms in China revealed by EST-SSR markers. Tree Genet Genomes 8: 205–222.

[42] Taniguchi F, Fukuoka H, Tanaka J (2012) Expressed sequence tags from organ-specific cDNA libraries of tea (*Camellia sinensis*) and polymorphisms and transferability of EST-SSRs across *Camellia* species. Breed Sci 62: 186–195. 23136530 10.1270/jsbbs.62.186 PMC3405963

[43] Wu H, Chen D, Li J, Yu B, Qiao X, et al (2013) De novo characterization of leaf transcriptome using 454 sequencing and development of EST-SSR markers in tea (*Camellia sinensis*). Plant Mol Biol Rep 31: 524–538.

[44] Wang XC, Zhao QY, Ma CL, Zhang ZH, Cao HL, et al (2013) Global transcriptome profiles of *Camellia sinensis* during cold acclimation. BMC Genomics 14: 415. 23799877 10.1186/1471-2164-14-415 PMC3701547

[45] Dellaporta SL, Wood J, Hicks JB (1983) A plant DNA minipreparation: version II. Plant Mol Biol Rep 1: 19–21.

[46] Thiel T, Michalek W, Varshney RK, Graner A (2003) Exploiting EST databases for the development and characterization of gene-derived SSR-markers in barley (*Hordeum vulgare* L.). Theor Appl Genet 106: 411–422. 12589540 10.1007/s00122-002-1031-0

[47] Charters YM, Robertson A, Wilkinson MJ, Ramsay G (1996) PCR analysis of oilseed rape cultivars (*Brassica napus* L. ssp. *oleifera*) using 5′-anchored simple sequence repeat (SSR) primers. Theor Appl Genet 92: 442–447. 24166269 10.1007/BF00223691

[48] Grattapaglia D, Sederoff R (1994) Genetic linkage maps of Eucalyptus grandis and *Eucalyptus urophylla* using a pseudo-testcross: mapping strategy and RAPD markers. Genetics 137: 1121–1137. 7982566 10.1093/genetics/137.4.1121 PMC1206059

[49] Van Ooijen JW (2006) JionMap 4, software for the calculation of genetic linkage maps in experimental populations. Wageningen: Kyazma B.V..

[50] Rabbi IY, Kulembeka HP, Masumba E, Marri PR, Ferguson M (2012) An EST-derived SNP and SSR genetic linkage map of cassava (*Manihot esculenta* Crantz). Theor Appl Genet 125: 329–342. 22419105 10.1007/s00122-012-1836-4

[51] Voorrips RE (2002) MapChart: software for the graphical presentation of linkage maps and QTLs. J Hered 93: 77–78. 12011185 10.1093/jhered/93.1.77

[52] ISO 14502-2 (2005) Determination of substances characteristic of green and black tea – Part 2: Content of catechins in green tea – Method using high-performance liquid chromatography. Geneva: International Organization for Standardization.

[53] Van Ooijen JW, Boer MP, Jansen RC, Maliepaard C (2002) MapQTL 4.0, software for the calculation of QTL positions on genetic maps. Wageningen: Plant Research International.

[54] Knott SA, Neale DB, Sewell MM, Haley CS (1997) Multiple marker mapping of quantitative trait loci in an outbred pedigree of loblolly pine. Theor Appl Genet 94: 810–820.

[55] Qin H, Guo W, Zhang YM, Zhang T (2008) QTL mapping of yield and fiber traits based on a four-way cross population in *Gossypium hirsutum* L.. Theor Appl Genet 117: 883–894. 18604518 10.1007/s00122-008-0828-x

[56] Li X, Wang X, Wei Y, Brummer EC (2011) Prevalence of segregation distortion in diploid alfalfa and its implications for genetics and breeding applications. Theor Appl Genet 123: 667–679. 21625992 10.1007/s00122-011-1617-5

[57] Zalapa JE, Cuevas H, Zhu H, Steffan S, Senalik D (2012) Using next-generation sequencing approaches to isolate simple sequence repeat (SSR) loci in the plant sciences. Am J Bot 99: 193–208. 22186186 10.3732/ajb.1100394

[58] Wang H, Jiang J, Chen S, Qi X, Peng H, et al (2013) Next-generation sequencing of the *Chrysanthemum nankingense* (Asteraceae) transcriptome permits large-scale unigene assembly and SSR marker discovery. PLoS ONE 8: e62293. 23626799 10.1371/journal.pone.0062293 PMC3633874

[59] Frenkel O, Portillo I, Brewer MT, Péros JP, Cadle-Davidson L, et al (2012) Development of microsatellite markers from the transcriptome of *Erysiphe necator* for analysing population structure in North America and Europe. Plant Pathol 61: 106–119.

[60] Bradeen JM, Staub JE, Wye C, Antonise R, Peleman J (2001) Towards an expanded and integrated linkage map of cucumber (*Cucumis sativus* L.). Genome 44: 111–119. 11269344 10.1139/gen-44-1-111

[61] Yu Z, Guo X (2003) Genetic linkage map of the eastern oyster *Crassostrea virginica* Gmelin. Biol Bull 204: 327–338. 12807709 10.2307/1543603

[62] Mano Y, Muraki M, Fujimori M, Takamizo T, Kindiger B (2005) AFLP - SSR maps of maize×teosinte and maize×maize: comparison of map length and segregation distortion. Plant Breeding 124: 432–439.

[63] Ferreira A, da Silva MF, Silva L, Cruz CD (2006) Estimating the effects of population size and type on the accuracy of genetic maps. Genet Mol Biol 29: 187–192.

[64] Behrend A, Borchert T, Spiller M, Hohe A (2013) AFLP-based genetic mapping of the “bud-flowering” trait in heather (*Calluna vulgaris*). BMC Genet 14: 64. 23915059 10.1186/1471-2156-14-64 PMC3751046

[65] Doerge RW (2002) Mapping and analysis of quantitative trait loci in experimental populations. Nat Rev Genet 3: 43–52. 11823790 10.1038/nrg703

[66] Kumawat G, Raje RS, Bhutani S, Pal JK, Mithra AS, et al (2012) Molecular mapping of QTLs for plant type and earliness traits in pigeonpea (*Cajanus cajan* L. Millsp.). BMC Genet 13: 84. 23043321 10.1186/1471-2156-13-84 PMC3504571

[67] Zhan H, Xu S (2011) Generalized linear mixed model for segregation distortion analysis. BMC Genet 12: 97. 22078575 10.1186/1471-2156-12-97 PMC3748016

[68] Sibov ST, de Souza CL Jr, Garcia AA, Garcia AF, Silva AR, et al (2003) Molecular mapping in tropical maize (*Zea mays* L.) using microsatellite markers. 1. Map construction and localization of loci showing distorted segregation. Hereditas 139: 96–106. 15061810 10.1111/j.1601-5223.2003.01666.x

[69] Alheit KV, Reif JC, Maurer HP, Hahn V, Weissmann EA, et al (2011) Detection of segregation distortion loci in triticale (×*Triticosecale Wittmack*) based on a high-density DArT marker consensus genetic linkage map. BMC Genomics 12: 380. 21798064 10.1186/1471-2164-12-380 PMC3156787

[70] Isemura T, Kaga A, Tabata S, Somta P, Srinives P, et al (2012) Construction of a genetic linkage map and genetic analysis of domestication related traits in mungbean (*Vigna radiata*). PLoS ONE 7: e41304. 22876284 10.1371/journal.pone.0041304 PMC3410902

[71] Petroli CD, Sansaloni CP, Carling J, Steane DA, Vaillancourt RE, et al (2012) Genomic characterization of DArT markers based on high-density linkage analysis and physical mapping to the *Eucalyptus* genome. PLoS ONE 7: e44684. 22984541 10.1371/journal.pone.0044684 PMC3439404

[72] Yin TM, DiFazio SP, Gunter LE, Riemenschneider D, Tuskan GA (2004) Large-scale heterospecific segregation distortion in *Populus* revealed by a dense genetic map. Theor Appl Genet 109: 451–463. 15168022 10.1007/s00122-004-1653-5

[73] Vogl C, Xu S (2000) Multipoint mapping of viability and segregation distorting loci using molecular markers. Genetics 155: 1439–1447. 10880501 10.1093/genetics/155.3.1439 PMC1461139

[74] Hackett CA, Broadfoot LB (2003) Effects of genotyping errors, missing values and segregation distortion in molecular marker data on the construction of linkage maps. Heredity 90: 33–38. 12522423 10.1038/sj.hdy.6800173

[75] Kenis K, Keulemans J (2005) Genetic linkage maps of two apple cultivars (*Malus*×*domestica* Borkh.) based on AFLP and microsatellite markers. Mol Breeding 15: 205–219.

[76] Collard BCY, Jahufer MZZ, Brouwer JB, Pang ECK (2005) An introduction to markers, quantitative trait loci (QTL) mapping and marker-assisted selection for crop improvement: The basic concepts. Euphytica 142: 169–196.

[77] Chen X, Zhao F, Xu S (2010) Mapping environment-specific quantitative trait loci. Genetics 186: 1053–1066. 20805558 10.1534/genetics.110.120311 PMC2975292

