# Supplementary figures and images for: Construction of a SSR-Based Genetic Map and Identification of QTLs for Catechins Content in Tea Plant (Camellia sinensis)
Source: PLoS One. 2014 Mar 27;9(3):e93131. doi: 10.1371/journal.pone.0093131 (PMC3968092; doi:10.1371/journal.pone.0093131)

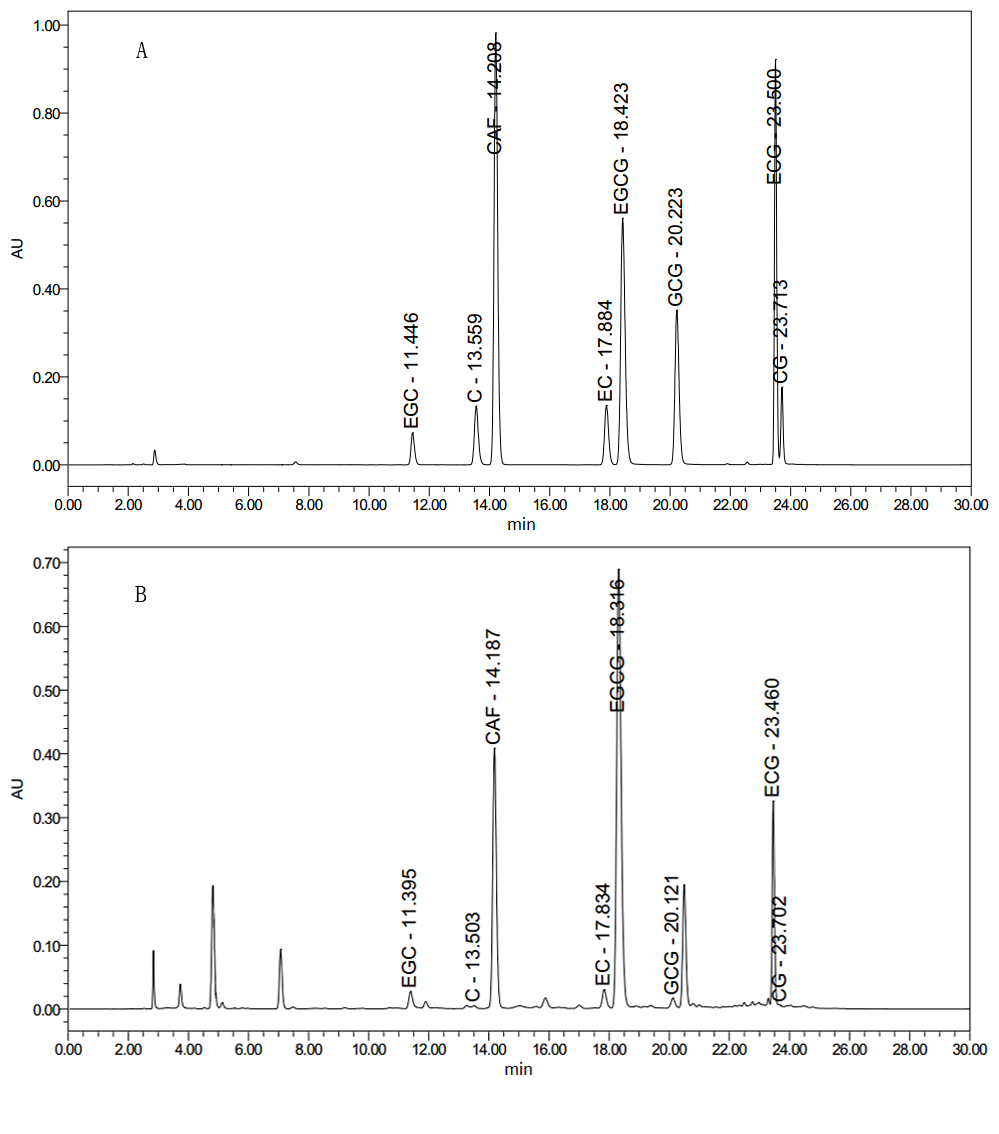

Supplement: Figure S1 — HPLC chromatograms of (A) catechin standard mixture, (B) tea catechins extracted from a typical tea sample (THB-010). (TIF) [file pone.0093131.s001.tif]

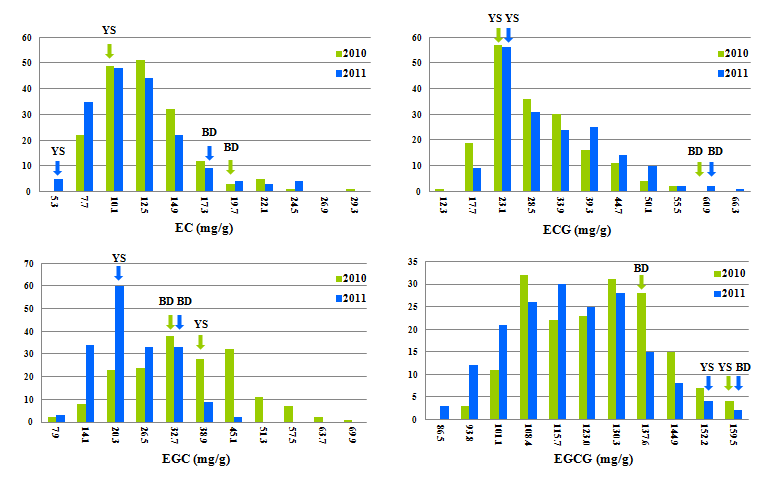

Supplement: Figure S3 — Frequency distribution patterns of catechins content in F1 population derived from the cross between ‘YS’ and ‘BD’. Parental values are indicated with arrows. (TIF) [file pone.0093131.s003.tif]
