# Supplementary material for: Construction of a SSR-Based Genetic Map and Identification of QTLs for Catechins Content in Tea Plant (Camellia sinensis)
Source: PLoS One. 2014 Mar 27;9(3):e93131. doi: 10.1371/journal.pone.0093131 (PMC3968092; doi:10.1371/journal.pone.0093131)
Supplement: Table S1 — Primer sequences and characteristics of the novel genic SSR markers developed from the transcriptome of tea plant. (PDF) [file pone.0093131.s004.pdf]

**Table S1 Primer sequences and characteristics of the novel genic SSR markers developed from the transcriptome of tea plant.**

| Marker ID | Repeat motif | Forward primer sequence | Reverse primer sequence | Ta (℃) | Expected allele size (bp) |
|---|---|---|---|---|---|
| TM231 | (TTTTC)6 | AGGAGGCACTGACTACA | TGGACCTATTGATACTTG | 54 | 229 |
| TM232 | (CTTTTTT)3 | CCACATCACAGCCAAAG | CAGCCCAAGCCTACCAG | 52 | 202 |
| TM235 | (AG)10 | GTTCTCATCACATCCTC | TACCATCAGTACATCTTG | 52 | 227 |
| TM237 | (GATGAG)3 | TTGGGAAACAAGAGTGA | TCTGGGACGAGGATAAT | 52 | 234 |
| TM239 | (TTCC)5 | AACAAGTAACAACACCC | TCCATATCCATCAATCA | 52 | 239 |
| TM241 | (GAGAA)3 | ATCGGCGACGGTGGAAGT | GCCAGCGGAGAGGAGAAG | 58 | 130 |
| TM242 | (AACCCT)3 | TGGAACCACTTTCGCCTG | TGAGCTCCCAACCATGAC | 58 | 107 |
| TM249 | (TA)8 | CAAGGTCCAAATACAAA | CCAATTCCCATACACTC | 52 | 122 |
| TM256 | (TC)12 | GAAACAGGCATTGAAGC | CTTAGGACCCAAAACAC | 56 | 121 |
| TM259 | (CCG)5 | GTGCGGCAAAGCTGTCTT | ACCTCCATCTCCAAACCC | 60 | 135 |
| TM262 | (CT)21 | CGACCAGACGGTGAAAT | AGGCTTGTGAGCAAAATC | 56 | 164 |
| TM313 | (TC)11 | TAAAACGATCACAACAC | GAAGGCTTACCCAAATG | 56 | 187 |
| TM314 | (GGT)5 | ACATGACCGGGACGAGA | CAAACCCTTCAGACATCA | 56 | 191 |
| TM315 | (TC)11 | AACCGATCAACCAATAT | ACCTCCGTTCAGCACTT | 48 | 164 |
| TM317 | (AT)12 | GAAACCTACACGCAACC | CCGGTCATCATAAACGT | 52 | 181 |
| TM318 | (AG)11 | CTGCTCCTCTTTAGTCC | AAATCCCTTTAACAGCT | 54 | 152 |
| TM319 | (AATG)4 | ATCATCATCGTCACAGT | CAGCATAAGTTGTTCAG | 54 | 139 |
| TM320 | (TTTTTC)3 | ATGGGACTCATCTATGGTAT | GCGTGGAGGCTCATTGGA | 54 | 285 |
| TM321 | (AG)12 | GAGGCACCTGTGACTTT | GTTGAATCCCCTTATGT | 52 | 195 |
| TM322 | (CT)13 | GCTTTTCCTGATTTGAC | TACCCTTTTATTCGTTTC | 48 | 250 |
| TM323 | (TC)10 | GGATTAAGCCCTGAAAG | GATGGCTATTCCGACAA | 56 | 174 |
| TM324 | (TTTTTG)5 | CATCGTTTCATTGCTTATT | ATTTTCGGCATTGTCTT | 54 | 176 |
| TM325 | (CT)17 | AATCTCAATACAACCGA | AACTCTTCTCCCTCCTT | 48 | 195 |
| TM326 | (TGA)5 | GGAACCCTTTGTATTTGA | ATTGTTGACCCTGATGC | 54 | 226 |

| | | | | | |
|---|---|---|---|---|---|
| TM327 | (TTTTA)4 | CAACAGAGGTGTAGGAT | TTCCATTCCCTTTTGAG | 45 | 143 |
| TM328 | (CCAA)4 | AGAGGTTGGGGCTTTGTA | ACCGACCAGTCCCTTTC | 52 | 163 |
| TM329 | (ACC)5 | AATGCCTTCCTTGTCCTG | TCTCCAATTCACTTCTCC | 52 | 138 |
| TM330 | (TC)10 | AATTAGAACCACCGCCA | CTTCACTCCCTCCCATCA | 54 | 122 |
| TM331 | (TC)10 | TTCCGGCGGTGTGTGTC | TCTCTCAAACTCGTCTCTG | 45 | 173 |
| TM332 | (CT)15 | TTATGTTTTGGGGTTTG | TATTCCCGTTTTGTTCC | 48 | 164 |
| TM333 | (TGAATt)3 | GAAGATTGTAGCCTCCC | GAGCCAAAGAGTATGTGA | 54 | 233 |
| TM334 | (CT)8 | GATTATTCCCAAAATGC | AGGCGGTATCCTCAGTG | 48 | 136 |
| TM335 | (CT)9 | TGCTGCTTGCAGTCGTG | CCCTTTCTTTCCCCTCT | 52 | 137 |
| TM336 | (TTTAT)3 | AGGCTTTGCTGCTTATT | GCTCGTAGTCATGTTGC | 54 | 169 |
| TM337 | (CCAATT)6 | GTGCGGCAAAGCTGTCTT | ACCTCCATCTCCAAACCC | 60 | 135 |
| TM338 | (ACA)7 | GCTTGCGGAACAACCTTT | GGCGTCTACATTCATTAC | 52 | 168 |
| TM339 | (GA)14 | CAACTGCCTACAACAACT | GATGCCTATCTCATACTAAC | 56 | 102 |
| TM340 | (ACC)9 | TGAGGACGACGAAGGAT | TCCGAGCCACCGAACAT | 52 | 113 |
| TM341 | (TA)10 | CATGCTCCCATCCCACCT | ATGCTGCTCATTCAAACCAACT | 58 | 111 |
| TM342 | (CAAAA)3 | GTCTGAAACCAAACCCAC | AAGGTTCTTCAAAGTGGC | 58 | 205 |
| TM343 | (TGTTGA)3 | ATCTTGGTAAGCTGCTCT | ATCATTGCTTTTGTTCTG | 56 | 179 |
| TM344 | (TC)11 | CTGGACTTCGATTCGGAGAG | AAAGGGTTCAACGCCTACCT | 52 | 138 |
| TM345 | (CT)12 | TTCTTGTCCTTCATGGAACTCA | GGAAATCTTATGGCACGGAA | 52 | 196 |
| TM346 | (AGAT)4 | GTTTCCAGTTCCAGATCCCA | TCCGCGTTTTCTTCTTCTGT | 52 | 232 |
| TM347 | (TTTG)4 | GTCTGGTGTGTCTCTGCTGG | CGCAAAACAAAGCCTAACTCA | 52 | 172 |
| TM348 | (TATC)7 | GAGATGGCTTGCTCAAGGTC | CCCCAACCAAATCAAATCAC | 52 | 275 |
| TM349 | (TTGGC)3 | AAAGCAGTGAGGAAGCCAAA | GGGGCCTATCCACCTATGTT | 52 | 217 |
| TM350 | (TGGTC)3 | TTCTTGTTTCTTCGGAGATCG | TGTCGCAGAATGATGGGTTA | 52 | 249 |
| TM351 | (GGAGAA)3 | GGGTGAGAGTAAAGGGGGAG | AAACACAAAATCAAATTTGTCAGAA | 52 | 247 |
| TM352 | (GAGGTG)4 | CTTCTTCCTGTCGGGTTGAG | GTCAACGGCCTATAACGGAA | 52 | 108 |

| | | | | | |
|---|---|---|---|---|---|
| TM353 | (CCGGCA)4 | GGAGATGTGGTGGTGAGGTT | CTCCACCTCAACTCTTTCGG | 46 | 241 |
| TM354 | (TCA)8 | TACCAACAATCTCAACGGCA | GCCACCAGAGCCATTAGAAG | 52 | 180 |
| TM355 | (CT)8 | TTTTTGCCCCAACAACGTAT | GCTTCCATTCCCAAATCTGA | 50 | 206 |
| TM356 | (TA)11 | AGGTTAGACGACGGCACATC | TACATCCCCATGCTCTCTCC | 54 | 245 |
| TM357 | (ATG)10 | GCCCTAACCACCAGATCAAA | GGGGAGTAGATGCGTTCTGT | 52 | 237 |
| TM358 | (CT)13 | GCCAGTGGCTCTTGACTACC | GCAGGTTTGAAGGGTTTGAA | 52 | 161 |
| TM359 | (TGT)7 | GCGGAGAGTTTCGAGTTTTG | CATCCATAGCATCATCACCG | 54 | 217 |
| TM360 | (CAT)5 | TTCAGACTGGTGATGCTTGC | GCTGCTGAAAAACAACCCAT | 52 | 134 |
| TM361 | (AT)9 | AGCGAAGAACAGGAAAGCAC | CTGAAAACTGCGATCGAACA | 52 | 273 |
| TM362 | (AG)12 | ATCTCCCTCTGGGTTCCATT | AGCAGTGTAGAGCCCATGCT | 52 | 161 |
| TM363 | (GA)9 | TTGTTTTTCTGGTGCAGTGG | CCAGAACGCTCTGTTTTGTG | 52 | 104 |
| TM364 | (ATC)7 | GACAGTCAAGCCTCCATTCC | TGATTGGAGAACACAACCGA | 52 | 116 |
| TM365 | (AG)11 | TCCCCCAATTCTCTGTTCAC | TCCTTAGCGCATCCATCTCT | 52 | 140 |
| TM366 | (AGA)5 | TTGGTGGGACTTCCAGAAAC | CTTTTTGGAGAGAGGCATCG | 52 | 228 |
| TM367 | (CTAGGG)3 | AGCCCTAGATTGGAGAAGGC | GCCCAATAAGGATACCCCTC | 52 | 205 |
| TM368 | (TGC)7 | ATTGCTGATACGATGGAGGG | ATCCAAAGAGGGGGCTTCTA | 52 | 200 |
| TM369 | (GAA)8 | CGGAGCTGGAATCTGAAGAG | GGAAGGGTTGCAAATTCTGA | 52 | 196 |
| TM370 | (CCA)5 | AAGAAGCAAATCTGCGAGGA | AGTCCATGACATCAAAGCCC | 52 | 214 |
| TM371 | (TC)9 | ACGCCACAGCTAACAAGTCA | ACCAAGCAAGTCCAAACCAC | 52 | 206 |
| TM372 | (TC)8 | CAAAAGAGGAGACGGTGGAA | TTTGAATGGCTGCAGAATGT | 52 | 158 |
| TM373 | (TTTTGT)5 | TTCATTTTCCTTGCGCTTCT | AAATTAGCCTCAGACCCCGT | 52 | 221 |
| TM374 | (CA)11 | TCATAAAAGGCCAGTCCACA | GCCAGAAACAGGTAATGGGA | 52 | 172 |
| TM375 | (AG)10 | CAACGACCTCTTCCTCTTCG | GTCGTAGCCGATGTAGCCAT | 52 | 200 |
| TM376 | (AGATGG)3 | GACGACTTGGAGGGTACGAG | CACGTATCCTTTGGCCTGTT | 52 | 231 |
| TM377 | (TC)8 | TTGCCAACTCTCAACGTCAG | AACGAGCGAGAGAGCAAGAG | 52 | 244 |
| TM378 | (AGGGTT)4 | CTCGGATAATGCATCGACCT | TTCACTTGCAATTCTCGCTG | 52 | 166 |

| | | | | | |
|---|---|---|---|---|---|
| TM379 | (AGA)5 | AATTCACCAAATCCCCTTCC | CTGTCTCTGCAACTGACCCA | 52 | 223 |
| TM380 | (TCAAGT)4 | TCAACACCTCCCCATCTTTC | TTGATCATGTTGAAGACGGC | 52 | 193 |
| TM381 | (TG)13 | GAGGGAGAGGATGGTGATGA | TCACCTTTCCACATTGTGATTC | 52 | 170 |
| TM382 | (CAT)6 | TCTCAAAACCAAATAGGCTCAA | TTGCGTTATGATTTCTGGGA | 52 | 162 |
| TM383 | (TC)9 | AAACCAAACAATTCCCACCA | CAAGTTCATTTGCTGACCCA | 52 | 190 |
| TM384 | (GGCCAA)5 | TAGACGTAACGCCGTCAACA | GTCGAAGGTACCAAGCCAGA | 52 | 197 |
| TM385 | (CCA)5 | CACCTTTATCCCCCGAAAAT | GGAGAAGGCTAGGGTTTTGG | 52 | 195 |
| TM386 | (ATTTGG)5 | GTGACTTCGAGAATGGCTCC | TCAACCCACAAACAAATCCA | 52 | 260 |
| TM387 | (TC)12 | TTATGCTGAGGAGGTGGGTC | CGGGCGATGTACACCTACTT | 54 | 220 |
| TM388 | (AG)12 | GCAATCATTCCATTCCATCC | AGGAGGAAGCATGCGATTTA | 52 | 260 |
| TM389 | (AGAAG)4 | TCCTTCAACATCGAACAGGA | TCCTCTGATTCCTTGCTGCT | 52 | 252 |
| TM390 | (TCT)7 | TTGTTCGGCAACAGAGACAC | TGGAAGAAGACGCACAAGAA | 54 | 254 |
| TM391 | (TCA)9 | TTTATCGACGGACGTTGGTT | ACAAGATTGACGATGACGGG | 52 | 163 |
| TM392 | (CT)15 | CTCCAATCCACCGTCGTACT | AACGGCAATCGGTAGAGAGA | 52 | 135 |
| TM393 | (AG)13 | GAGATGACATTTCGTCGGGT | TTGTGGTTATTCCTTTGGGG | 56 | 272 |
| TM394 | (AGA)8 | GGAAGCGCTTAGACATGGAG | CCACCTTTCTCTCTCCCCTT | 52 | 245 |
| TM395 | (TCTTTT)4 | GATTGTAGGACAGCCGTGGT | AAGTTGGGGCTTGTTAAAGGA | 52 | 266 |
| TM396 | (GA)9 | GGAATTTGTGGGAATGATGG | AAAGACCAAACCCAAACACC | 50 | 178 |
| TM397 | (CAT)6 | CTCAGCATCATCCCCAGAAT | TGACGAAGATGACGACGAAG | 52 | 127 |
| TM398 | (TCTTGC)3 | CCGAATTGGTCAATGAGCTT | GCGAAGGATGGAGGAGTAGA | 52 | 131 |
| TM399 | (TC)8 | TCTCTGCTCTGCTGCTTTCA | CGATCTGGTTGGGACAAAGT | 54 | 107 |
| TM400 | (ACC)7 | CTCCTCTGTTTACGCCTTGC | CCGCCTGTATTATCCCTTGA | 52 | 162 |
| TM401 | (ACC)5 | TCAAGGGATAATACAGGCGG | TTTGTGGACTCTTGTGGTGG | 52 | 263 |
| TM402 | (GA)10 | TGTCCAGAACAACAGGACGA | GAGATGAGAAGAACTCGGCG | 54 | 216 |
| TM403 | (AAAAC)3 | CGTATCGCCGTATCCGTATC | GTGTGGTGTGATTTTGGCAC | 52 | 202 |
| TM404 | (AG)10 | TGCATTTGGTTTGGTTTTGA | TCGCCAGTACGATTCAACAG | 52 | 133 |

| | | | | | |
|---|---|---|---|---|---|
| TM405 | (AG)14 | TTGAGGGGTTTCTGATCACC | TGGTCAAACCAACAAAGCAA | 56 | 225 |
| TM406 | (AAAGA)5 | TTTCCCCACACAGCTACACA | TTTCAGGTAAACCCCACTGC | 60 | 181 |
| TM407 | (CAAGAT)3 | AACAACAGCAGCGAAGATGA | CCACCACTGATGACCCTTTT | 52 | 251 |
| TM408 | (AG)12 | AATGGCGTTGTGAGCAGAC | ACCAGCAAAACAAGTCGTCC | 52 | 279 |
| TM409 | (CATCTC)3 | ACGGCTTTTTCTGTGGTCTG | GTGCACTACTCTCAGCAGCG | 52 | 193 |
| TM410 | (AG)8 | TCACGAGATCCCCATCTTTC | AATGGGTTTCTGAGAATGCG | 54 | 239 |
| TM411 | (AG)14 | TTCCGTCGTAACATCCACAA | GAGTTGCGAGGAGAGGAAGA | 54 | 196 |
| TM412 | (AG)14 | GCAGGCTTCAAGGTTTTCAG | TGTTGTTCTGGCGTTGAGAG | 52 | 215 |
| TM413 | (CT)8 | TGTGGCCTAGTTAACACCTCC | CAAAGTTCGAATTCGTTCGC | 56 | 248 |
| TM414 | (TCAAGC)3 | TACAGCCACCACACCAAAAA | TGTTTTCCACTGAGCACTCG | 52 | 257 |
| TM415 | (CCTTC)3 | TCCACCCAAAACCTACTCTCTC | TATTTCGGAAACGAGCCATC | 52 | 125 |
| TM416 | (TC)11 | GAGGCATGTGGTGTGTGAAC | CTTGCATTGGGTTGTGTGTC | 52 | 199 |
| TM417 | (CT)17 | GCATAGCAGGTGCATGAAGA | TTTGGTTCATTTCGGGACTC | 50 | 190 |
| TM418 | (CT)11 | CATGGGCCTCTCTGTCAAAT | AGTTTCATCGATGTCCCAGC | 52 | 219 |
| TM419 | (AG)11 | GGAAACACATCCACTCCAGG | CGTTTCTCTGATTCTTCCGC | 52 | 135 |
| TM420 | (CACGGG)4 | GCCAATGCAGCATCTGATAA | AGTTCTGTTTGCAGGCTGGT | 52 | 247 |
| TM421 | (CT)11 | TCTATCGATCTTTGCGGCTT | GAGAGAGGGGAGAGGAGAGG | 50 | 220 |
| TM422 | (TTC)7 | GGACTTCGTTGCTTCCTTTG | CCATTCTCGACGAATCCAGT | 52 | 167 |
| TM423 | (CT)12 | CTCCTCATCCGACGATCATT | GATCGATCGAGGGAAATTCA | 56 | 144 |
| TM424 | (GA)9 | TAGATCATTCCCGCAACCTC | TCTCAAGACAGGCAGTGTGG | 52 | 276 |
| TM425 | (TTATT)3 | CACGTTCGCATATTTTGGTG | TTGCTGACGACAACATTTTATT | 52 | 225 |
| TM426 | (AGA)11 | TGAGAGTGCTTGTCTGGGTG | CAACTACCCCTTTTCCCCAT | 52 | 245 |
| TM427 | (ACCCTC)4 | TCCAACCATGTATTCCCGTT | GATTAGCGAGAGCAGAAGCG | 52 | 138 |
| TM428 | (CAC)7 | TCTCCTCCTCGATCCTCAGA | CCCTCTTCTTCGGATCCTTC | 52 | 195 |
| TM429 | (AG)8 | CCTATATAGAGGCGGGACGA | TGAACACGGTGGTAATCGAA | 52 | 134 |
| TM430 | (AG)9 | AACAACCAATGCAATCCTCC | GAGTGATCGCCAGCTGTGTA | 54 | 180 |

| | | | | | |
|---|---|---|---|---|---|
| TM431 | (AAT)6 | CCTCCTCCTTACCCATCCTC | TGTGGGTTCAATTTTCCCAT | 52 | 252 |
| TM432 | (TC)12 | CTACCAGAGCGAAACCCTTG | CATAGAAATCAAAACCGCGA | 52 | 135 |
| TM433 | (ATAG)5 | TACACACACCCCCACTCTCA | ATCGTTCGCTCTGCTTGAAT | 54 | 233 |
| TM434 | (CT)9 | CTGTGTGAAAGAGCAGAGCG | TAAAACCCGGTGGCATTTAG | 54 | 135 |
| TM435 | (CTC)6 | GGTGGTGTGGTTTAGAGGGA | TTCATCTTGGGGAGCAATTC | 52 | 242 |
| TM436 | (GATTCA)4 | AACCCATTCTTTTCCGAACC | CAACGAAGGCAATGTCCTCT | 52 | 273 |
| TM437 | (AG)18 | CGACAAGGTGGTTGATGATG | CCATTCTTTTGGTTGAGGGT | 54 | 269 |
| TM438 | (CT)11 | ATTCCCAATCCTCATCCTCC | GGTGGAGGAGACTTGTTCCA | 52 | 149 |
| TM439 | (AGAAA)4 | CTGGGAAAGCTGAAAGTTGC | TTTGGCATTTGTTTTCCCTC | 58 | 189 |
| TM440 | (TTTGC)3 | TTGACCCGAATAAAATGGGA | CCTCAAAACATGCTTTTCTTAATC | 52 | 159 |
| TM441 | (TC)8 | CCTTCCTTGCTCTCAAATGC | CGGGAATGTTGTGTTTGTTG | 54 | 178 |
| TM442 | (ATACAC)3 | CAAGCCAAACCTTGCTGAAT | CTGTCCTGTGTCTGGTGGTG | 52 | 275 |
| TM443 | (TC)14 | TCTCTTCCAATCCCACCAAG | CTGCTCTGGGCAGTGATTTT | 52 | 139 |
| TM444 | (TCA)9 | TTGAAGCCCTGCACTAACCT | GGTGGGAAATTGAGGGATTT | 52 | 103 |
| TM445 | (GTA)5 | CCCAAATCCCAAGCTGTAGA | ACGATCGAGCCTGCAATACT | 52 | 261 |
| TM446 | (TC)13 | GTCGCTCTCTGAGATGGAGG | GCACGGAGAGATTCGAAGAG | 54 | 126 |
| TM447 | (AAAAG)5 | TGTTGTTAACGGTGTTCGGA | GCATTTGTTTTCTCTCTCTGCC | 54 | 156 |
| TM448 | (CAC)5 | CAGTCTCCTCTGCAACACCA | AAAGGTCGAAGTGGGAAACC | 54 | 108 |
| TM449 | (AG)12 | TTAGATTCGCCCCAACAAAC | CCTCCCCTGTTGTTATTCCA | 54 | 172 |
| TM450 | (AG)11 | GAATTGTTCGATGACGCAGA | ACCACTCAATCCAAACGGAG | 52 | 209 |
| TM451 | (GA)9 | AACAGCCCTGAAATTGGTTG | ACCCCTTCCACATTCTCTCC | 56 | 261 |
| TM452 | (AG)18 | AGGATCGTCAACAGTACCCG | TGCAATCAACCACACCTGTT | 56 | 163 |
| TM453 | (TTC)6 | AAGTCACAACACCACCACCA | GAGGCAGCGATAGTACCAGG | 52 | 268 |
| TM454 | (AT)9 | TTTCAGTGGCACTTTTGGGT | CGGTTCATAGCAAAATGAGGA | 54 | 182 |
| TM455 | (TCG)6 | ATCGCTTCAGTTCCTCTTCG | GAGGACCTAAATCCGAAGCC | 52 | 216 |
| TM456 | (AAAGA)3 | AGGTGTGCTTGGAATCTGCT | CCCCTACACGTCTGTGGATT | 52 | 229 |

| | | | | | |
|---|---|---|---|---|---|
| TM457 | (TTAGGG)5 | AGTACCGACAACACTTCCGC | AACTTCCCCTTTCCCTCCTC | 52 | 186 |
| TM458 | (CT)8 | CCCAAAGAAAATTATGCAATGA | ACCTCTTGAACAGAGCCGAG | 54 | 125 |
| TM459 | (GAT)5 | CGGGTGCCATTGTACTTCTT | AATCAGCATCACCATGAACG | 52 | 279 |
| TM460 | (GAATT)4 | GGAGATCAGATCGAATGGGA | TCATCAAGGGAGAACGAACC | 54 | 156 |
| TM461 | (ATTTTT)6 | GGCTAGGGTTTCTCCCACTT | GAAGGTCGAAGCGATGTTGT | 52 | 211 |
| TM462 | (AC)8 | ACAACCCAACCACCATTGAT | GTGAACAGGCTTGACAGGGT | 52 | 194 |
| TM463 | (AAGAA)3 | CACTGGTACCACGGGAAATC | TTTGATGGAAGAGGACTGGG | 52 | 114 |
| TM464 | (ACCA)5 | TGGCATGTGACCAAAAAGTC | TCAGCAACCCAAAACACTTG | 52 | 155 |
| TM465 | (ACC)7 | GCATAAAGTCCAAAGCTCGC | GGTGGGTATTGTGTTCTGGG | 52 | 201 |
| TM466 | (GATGAG)3 | AGTGGTTCCGACAAATCAGG | ATTGCCTCAGCATTGGATTC | 52 | 204 |
| TM467 | (ACAAA)3 | TGAGAGAACCCATTTGGGAC | GCCTTTGGAGTTGAGAGCAC | 52 | 262 |
| TM468 | (CAC)5 | CTTGGCTCCTGCAATCTCTC | GCCTTTGGAGTTGAGAGCAC | 52 | 197 |
| TM469 | (CT)14 | TCAAATTGACTCCACACACACA | ATTCTTGGGGAGAGAAGGGA | 50 | 174 |
| TM470 | (GAA)6 | AACACCACTCAAAGCGACCT | GGAAGAGGATCGAGAAACCC | 52 | 279 |
| TM471 | (CTC)5 | ATGTATCGCTGTCTGGGGAC | CAAGAAGAAGCCTGACCCTG | 52 | 138 |
| TM472 | (CCATTC)3 | CAAGCGTAGCTCCTTCCATC | AAGAGGGGTTGAGGGAGTGT | 52 | 192 |
| TM473 | (AGG)5 | CCGGTGTGGGAGATAAAGAA | ACTCTCCTTGCCCTCCTCTC | 56 | 152 |
| TM474 | (TC)8 | TTTTCTGGAACGACCAGGAC | GAGGAATGAAAGGTGGGGAT | 54 | 228 |
| TM475 | (ATCAA)3 | TCATTCCAATGCTGTCCAAA | GCTCAACCCGTCTCTCTGTC | 58 | 253 |
| TM476 | (CAC)5 | AGCCCGCTAGCACTCATAGA | TGCACGAGACAACAAAAAGG | 52 | 220 |
| TM477 | (GGTTAG)6 | TTGAATTTGGATCGGGGTAA | ATCAAATCAACCCCCTCCTC | 52 | 161 |
| TM478 | (CTCAGG)4 | TGCTTGCCTATGGTTCAAGA | ACAGAGGAAGAAGCATTGGC | 56 | 270 |
| TM479 | (GAA)6 | ATTTGCGGTTTGGAATTGAG | AACAACCACCATTCCTCTGC | 52 | 239 |
| TM480 | (GTA)5 | CGAAGAGTCGTTTCGAGGAG | CATCCCTTGTCTTCTCCCCT | 52 | 208 |
| TM481 | (CT)16 | CTTCCAAAATCCTCGTTCCA | CTGTTTTGGGCGTAACGATT | 52 | 105 |
| TM482 | (TCTTC)4 | AAGAGAAGCTCCCACCATCA | CCAAGTTTTGTGTTTCATCCC | 54 | 279 |

| | | | | | |
|---|---|---|---|---|---|
| TM483 | (AG)12 | GGCTAGTCCGTTGGTTCAGA | TTGTGAACTTGATCGCGAAG | 50 | 133 |
| TM484 | (TG)10 | CGGAAATGCTCGGAAAATAA | TCCACCCTGTTTTGCTTCTC | 50 | 227 |
| TM485 | (TTTC)4 | CTCCAGTGGATGTGGATGTG | AAAAGGCACCTCAACAAATG | 52 | 224 |
| TM486 | (TCA)6 | ACACAAATAACCAAGCCCCA | TGCATAAGCTTGATGGCTGT | 52 | 242 |
| TM487 | (ACCT)5 | CGACAAGTCATTCAGCTCCA | GTGGCTTCATTGGGAATCTG | 52 | 111 |
| TM488 | (AAC)6 | CACTTCAAATCCCCCAGTGT | AGCTCCATTAAGGGTTCGGT | 52 | 263 |
| TM489 | (ATG)8 | CAGCGACAGAGGTGATGAAA | CTCCGGCAGTTGAATCTCTC | 52 | 201 |
| TM490 | (CTACCT)5 | TGGGCCAGAAGAGAAAAGAA | GGTGTTCCTGGCACTTCAAT | 52 | 177 |
| TM491 | (AG)14 | GCCCAACATACAGGTTGCAG | GGGGCGGTTTTTGTATAGGT | 52 | 172 |
| TM492 | (TTTTTA)3 | TTCCAAAAACATGTGAGGCA | CCAGGGGATGCAATTAACAA | 52 | 256 |
| TM493 | (AGG)6 | GATAGGGACAGAGATCGGCA | TTTCCAACCTTGCTCAAACC | 52 | 242 |
| TM494 | (ACC)9 | AAATGCGACTCCAGCTCACT | TTTTGGGAGGTCCAGTTTTG | 52 | 224 |
| TM495 | (CT)10 | GGATCTCCATGCTAAGCTGC | CCACATTTCAAGACAGAATTGC | 52 | 229 |
| TM496 | (GA)9 | TGTTGTGACCTTCTCAGCCA | GGATCCGGACTCAGTTTTGA | 52 | 228 |
| TM497 | (TTCCAC)4 | TCGTATCTCCCCATCTCGAC | CGATAGGCACCGATTCATTT | 48 | 273 |
| TM498 | (CTAACC)4 | GCGCAGAAATCAGGAAAGAC | GTTTTGGGGAAGAGAGGAGG | 58 | 272 |
| TM499 | (AGA)5 | AACTGTGACACCGATTGCAG | AAGTTTCACTTGCCAGCACC | 54 | 255 |
| TM500 | (GA)18 | GGCGTCTCTCGAAGAAATTG | AACCGGACGTCAACAGATTC | 54 | 219 |
| TM501 | (GAAAG)3 | AAGCGCAGAAGAAAGCTCAG | ACTGGATAATGCCAACCCAC | 54 | 258 |
| TM502 | (AGAT)4 | TGTCTTTTGTGGTTTCGTGC | GGGAGACGATGGATCAGAAA | 50 | 177 |
| TM503 | (CT)8 | GGGAGGGAGGAGTATCTTGC | ACTTCATCAGTGGGCTCCAT | 50 | 136 |
| TM504 | (AGG)8 | CCATGCTTGTTGAGAGCGTA | TTGGGAAGAGTCTGGGAAGA | 52 | 277 |
| TM505 | (TTCCC)3 | AGCCAAATTACGACGTTTCG | GTTGCTGCTTCGCCATTAAA | 52 | 266 |
| TM506 | (TC)8 | CTGGTGATGATGAGCGAAGA | CACCCCTATCTGCTTTTGGA | 52 | 224 |
| TM507 | (AAGCGG)4 | ATTGTCACCGGAGTTTTGGA | ATTTGGGAGTTGGCAGAATG | 52 | 260 |
| TM508 | (AATAGC)4 | CAAATGCATAATGTGGTCGC | ATTTCCCTCCCTTTCATGCT | 52 | 215 |

| | | | | | |
|---|---|---|---|---|---|
| TM509 | (GA)8 | CCTCCTTCCATCATCCTTGA | TGGAGATAACACCCATTTTGG | 50 | 131 |
| TM510 | (TC)11 | TTAGGCAGATCGATTCCCAC | AACCAGAGCCAGGAGGAAGT | 52 | 164 |
| TM511 | (TCT)6 | CGTGACTAGCCACAAACCAA | TGCTGACTTGACCTTGCATT | 52 | 277 |
| TM512 | (CTC)6 | AGTCAAACCTCTCGCTTCCA | AGTGTCGAGGAGGTCGTGAT | 54 | 200 |
| TM513 | (AG)10 | CAAGCGATCAACAACAATGG | TTGAGAAATCAACCCCTTGG | 54 | 265 |
| TM514 | (TCA)5 | ATGTCTGGCCGTGGATTAAG | ATGGCAGGCTGTTCTGATTT | 52 | 257 |
| TM515 | (CAA)6 | GGCTACTGTCTCTGCCCTTG | AACCGAGTAGTGAGAGGCGA | 56 | 274 |
| TM516 | (GGATT)4 | GCCTGCCATTGATGAATTTT | TCGTCTGCTTTTCTCTACCCA | 52 | 143 |
| TM517 | (GAG)6 | GACAATCCTTCCACTGCGTT | TTCCTTTGAGTGTCCTTCCG | 54 | 242 |
| TM518 | (AACAAT)4 | TCATCTTTACACCCTCCCCA | TTGGTTCTTTTGGGTTGAGG | 52 | 278 |
| TM519 | (TCT)5 | GCCTCCGACGTGAAGATTAC | CGCCATGGTTGAGAAGTAGG | 52 | 269 |
| TM520 | (AGGAAG)4 | AAAGTGGACCTGAATGACGG | GCTCCACTTTCGACACAGGT | 52 | 172 |
| TM521 | (AG)8 | GAAGGTGGAACTCCGATCAA | TGTCCACATACCTCAACCCC | 52 | 247 |
| TM522 | (TC)15 | AATTGGTGGAGAGATCGACG | CAGCTCTCTCTTCCACTGCTG | 60 | 217 |
| TM523 | (AAAAGA)3 | TTTGCATTTTTGCCAAGTGA | CTTGCGTGACAATGCTCATAA | 52 | 276 |
| TM524 | (CAAACA)3 | GAGGGGATTTGGATTTGGAT | CAAAGAAGCACAAAGAAAAAGAAA | 52 | 231 |
| TM525 | (CAA)8 | TTCAATCCCTTGAAAATGGC | AGAGGGGTTGGAGATGGAGT | 56 | 171 |
| TM526 | (TGT)8 | CGCCATTGATAGGTTCCAAA | AGGAAAGGTGTGCAGATGCT | 52 | 277 |
| TM527 | (GA)12 | GGGATGGATAGATGATGAGGA | ATGACGATTGACCACTTCCC | 56 | 123 |
| TM528 | (TTG)7 | TCTCTCATCTGCGTCCCTTT | ACAGTAACACGGGTGGCTTC | 52 | 245 |
| TM529 | (AG)8 | GAAAATCACTCAACGCCACC | ATAGTACTCGTGGCCGATGG | 52 | 246 |
| TM530 | (TCA)7 | CCGTGTTTACCACCACCCT | CCCTGGGAACAAGAAAGTGA | 52 | 273 |
| TM531 | (TAG)5 | GGGGATTCTGATGAACTCCA | CCACATTCTCTCACTTGCCC | | 159 |
| TM532 | (TTC)7 | TTCAAAGTCATGACAGCCAAA | GCCACTCCTCCAACTCCATA | 52 | 179 |
| TM533 | (GA)9 | CCGTAGCCTAATACATTGTGCAT | CGGGCAATCTTTCAATTGTT | 52 | 234 |
| TM534 | (TA)8 | AGCAGAGTTGTGTGCGTGTT | GCTCTGTGTGCTTTGGATCA | 52 | 233 |

| | | | | | |
|---|---|---|---|---|---|
| TM535 | (AAAACA)4 | TCAATCACCCTCCATTGAAA | CGTATACCATGGTCGGAAGG | 52 | 190 |
| TM536 | (TTTGG)4 | GTGGCTTCAAAGAACAAGGC | AACGAGTTGACTGACCCCAC | 60 | 206 |
| TM537 | (CAAGTC)3 | GAGGGCCAATAGTGGAGTCA | AAAATGGCACCTTCCAACAG | 52 | 179 |
| TM538 | (AG)9 | AAAATTTTCTCTCTGCACAATCG | ACCCTCCTTGTACCCCTTTG | 52 | 194 |
| TM539 | (CAATAC)3 | GATTTTGGCTCTGCTTCCAA | ACCTGCACCGCACAAGTTAT | 52 | 129 |
| TM540 | (CCA)6 | CTAGTGTTGGCAAAGTCGCA | GGCTGAAAGATTGGGTGTGT | 50 | 151 |
| TM541 | (TCT)6 | AACGCACGAGGAGAGACACT | GAGAGAAGCGGAAGCAACAC | 52 | 160 |
| TM542 | (TGA)8 | GCAGCGACAGTTCTGTTGAA | CACCACATCCCCTTCCTCTA | 52 | 245 |
| TM543 | (TAAA)5 | AGGTGGCCATACCTACGTACA | TGGAAGAGTGCATTGCATGA | 52 | 201 |
| TM544 | (GT)8 | GAGGGCCAAAACTGATGTGT | ACTTCTGCTGCAACAAACCC | 56 | 174 |
| TM545 | (GT)8 | AACGGATCTTTAAGACGGCA | CCACCACCTTCTAGAACACCC | 52 | 178 |
| TM546 | (ATCTCA)3 | TTGAAGTTAGCCAAGGCATC | TCCCAATCCCTTTAGTGTGC | 52 | 115 |
| TM547 | (TAGGAT)3 | GAGAGACAGAGAGATGGGCAA | TGACCACCACCAGACCACTA | 54 | 108 |
| TM548 | (AGG)6 | GTGAGGTAGAGAGGGAGGGG | CCTGCAATTGCCTCCTCTT | 52 | 217 |
| TM549 | (CCAACT)3 | TGTTCTGCTGTGTTTACCGC | TGGCTGGAATATGGTTCTCC | 56 | 259 |
| TM550 | (GTC)7 | TCGCGTTAGGGTCTCCTAGA | CACAACAAGTCCATTGTCGG | 52 | 184 |
| TM551 | (CATCAG)3 | TCCGATCTTCATTCCTCACC | AAGAGGGGTGGGTTGAGACT | 52 | 127 |
| TM552 | (GTTGG)3 | TTTTTCAGCGACAGCTCAGA | GTGTTTCTTCGCGTGTCAGA | 52 | 190 |
| TM553 | (AGAAG)4 | CTCTGAGGCAATCTTGAGGG | TGAGATCCTAGTGTTCCATCACA | 52 | 157 |
| TM554 | (AGATGC)3 | CGAGTGAATATGGACAGCGA | CAAGATGCCCCCTATGAGAA | 52 | 212 |
| TM555 | (GA)10 | TATGGGTATGGGTCTGCACC | GAGCCCTCCAACCATCATTA | 52 | 117 |
| TM556 | (TATTGT)3 | TTTTTCCGATATGGAGGCAG | CCCCACAGCCCATTTATCTA | 52 | 213 |
| TM557 | (AGA)5 | AATCAAAACTCCCCAGCAGA | TCAGCACTCACAGTGAACCC | 52 | 101 |
| TM558 | (CAAAGA)3 | GAGGTCTCCGAAGCTTTTCC | GAGGAGCGAGAGATGGGTTT | 52 | 130 |
| TM559 | (TA)12 | TGTTGTGATGGACCTGGAGA | GGGAAAACTGGAAAACTTCAAA | 54 | 230 |
| TM560 | (TC)10 | CAAGTTTGCGAGTCCTCCTC | GCCCACGAGGTTAGATGTGT | 58 | 185 |

| | | | | | |
|---|---|---|---|---|---|
| TM561 | (CAA)6 | AACCCAGCAAACAAAACCAG | TGGACTCTGAAGGTTTCTCCA | 52 | 226 |
| TM562 | (TATCA)5 | GTCCGGCAAGATTGGATTTA | TCGGTCGATCATCACTGTGT | 52 | 169 |
| TM563 | (TTTTTG)3 | TTGCGATAACATCCCAGTGA | TTTTCATTTGCCATCACCAA | 52 | 243 |
| TM564 | (TC)9 | AATTAGGGTTTCCCACCTGC | CGAAAAGATCACAACGCTGA | 54 | 190 |
| TM565 | (TCCGAA)3 | TTCCTCTTCAACCCCAACAC | GAAGTTCGCAACGATTGGAT | 52 | 252 |
| TM566 | (ACC)7 | ACAACCTCTTTCTAGCCAAGTCT | ATGTAAGGCCAACCCATTGA | 52 | 207 |
| TM567 | (GA)12 | CCCCTCTTCCAACTCCAAAC | CCAATCGACCTTGTAAATAGCA | 56 | 274 |
| TM568 | (GA)8 | TAACCGCGGAAAATCAATTC | GCCTGATGGAGCTCTCTTTG | 52 | 196 |
| TM569 | (GTGA)5 | GCAAATTCGTAAGGCGAGAG | CTGACGTTTACCCTCGTTCC | 52 | 274 |
| TM570 | (ATTCG)3 | CCAGTCCGCAATCGAGTAGT | AAGGAGGAGTGAACTGCGAA | 50 | 216 |
| TM571 | (ACA)5 | ACGAGTTCGAACCAGACGAC | CGCCACTTGAATGGGTAGTT | 52 | 251 |
| TM572 | (AG)35 | GTCAAGCATGGGAGCTTCTC | TTGTGAATTTAGGCGAACCC | 52 | 234 |
| TM573 | (TC)8 | TTCACCGGCTCTTTCTCACT | ATTCATCCCTCCTCTACCCC | 56 | 172 |
| TM574 | (CAT)5 | GTGTGTTTGAGACTCGGGGT | TCCGTGACTGATTTATGTGTTCA | 52 | 228 |
| TM575 | (TCATC)5 | CGGCGAAGAGAGAGAGAAGA | CTGTTGTTGTTGCTGCTGCT | 58 | 211 |
| TM576 | (TTTTC)3 | CGCTCTTCCTTGTTTTCTGG | CACAAGCCATTGTAGAGAGAGAAA | 52 | 223 |
| TM577 | (TTG)5 | ACTGCTTCCCCTTCTTCCAT | TTCTTCCCACCTTTCCTTCC | 52 | 238 |
| TM578 | (TCCTC)3 | ACCCACGAGGTCACGAGT | CGGAGAGGCTTGAAAGAGTG | 52 | 187 |
| TM579 | (AAAAG)5 | TCCTTCATGGAGATTCCACC | AAACGAACAAAAACTAGCATCCA | 52 | 250 |
| TM580 | (GTTGCT)3 | ACTCTGGAGAGGCGAAACAA | CAACATCAACATCAGCAGCC | 52 | 234 |
| TM581 | (AAAAAC)3 | AAGGATCACTGGTAAAAAGCCA | CTTCTGAGCCGTTCTTGAGC | 52 | 228 |
| TM582 | (GGA)5 | GAACCCACGACGAAGATGAT | CTTCTTCACACCACCCCAAT | 52 | 279 |
| TM583 | (TTTG)4 | GCGACTGTTTGAAGCAATGA | GGTCGAGAAGAGGATGGTGA | 52 | 234 |
| TM584 | (GAAGAT)3 | GACGGAGCTCTCGAACAATC | TCTTCGCCTTTGTTTTTGCT | 52 | 166 |
| TM585 | (TAAAAA)3 | GCTCAAACCAACTCAAAAGTTAGAA | TTTCCCCATTGATTGTGTCA | 52 | 196 |
| TM586 | (TGAGAT)4 | TCGATAATACCCCCAACCAA | TCTCTCTCAAAAGTGGGCGT | 52 | 186 |

| | | | | | |
|---|---|---|---|---|---|
| TM587 | (GA)9 | GCGAGATTGGAGGAGAAGTG | TTTCTCCCTCTACCCAAGCC | 52 | 277 |
| TM588 | (CT)12 | TGAATTTCTGTTTCCCCAGC | ACTTTCAGCTTTTTCGGGGT | 52 | 236 |
| TM589 | (CTCCT)3 | CACCACTGCCCAACAAACT | GAGGATGATGATTCGGGAGA | 52 | 211 |
| TM590 | (CAAAAT)3 | ATCTACAAACCCACCTCCCC | GAGGATGATGATTCGGGAGA | 48 | 145 |
| TM591 | (TCA)5 | GACCCTGCCCTCCTTAACTG | CAATTCAACACTTCCAGCCA | 52 | 269 |
| TM592 | (GA)8 | GCAGAGACTCCGAGAACCAC | CCCAGAGCATTTCCAACAAC | 58 | 170 |
| TM593 | (ACACA)3 | GGTGCACAAGTCCAGTTCCT | TGGAGCAATATTTGTTAGCCTC | 52 | 236 |
| TM594 | (GA)12 | GTGCGGTTGTAGACGGAGAT | CCACCGGTATCAACCTACCT | 52 | 274 |
| TM595 | (CT)8 | GCTTCTTTTGGGCTGTTCAC | TGTGGGTTTGCTGTGATTGT | 54 | 150 |
| TM596 | (TCGAA)5 | CGTACTTCAACGCTATAGCTCTCT | CTTCGGCATGGCTTCTAAAC | 52 | 198 |
| TM597 | (CACCAT)3 | CTCATGGGCAGACTCCAAAT | GTAGCGGTGTCGAAGAGGAG | 52 | 116 |
| TM598 | (AAT)5 | GTACTCTCCCACTCGGAGCC | TTGACATTGCTCAAACCTGC | 52 | 132 |
| TM599 | (CT)10 | GCGAAACCACTGGATCACTC | AGCTTTGGCCAGTCTTTTGA | 54 | 134 |
| TM600 | (AAAAT)3 | TTTTACGGCCTGAAAACGTC | TGTTTCTGCACCACCACAAT | 50 | 182 |
| TM601 | (GGA)5 | TTGCACTGGAGTGCGATAAG | CATCGCCACCAAACTCTTCT | 52 | 276 |
| TM602 | (TCTGGT)3 | CAGCATCCAATGTCTCCTCA | GGGCTGTTGAAAGCACAAAT | 52 | 255 |
| TM603 | (TC)10 | TGTTTCCCATGCTATCCCTC | GACACAAACCCTAACCCCCT | 56 | 100 |
| TM604 | (TTTG)5 | TCTCAATCAAGGTTCCGAGG | TGACATATTCGCCCAACAAA | 52 | 196 |
| TM605 | (ACA)5 | CAATGGGTACACCAACAACAA | CCCTGCTGCCTCTCATACTC | 52 | 279 |
| TM606 | (CT)11 | TCATCGGCTGAAGATCAAAA | TCAAGCTCCTCCTCGTTGTT | 52 | 177 |
| TM607 | (CTCAAG)5 | CAGCAACAACAATCATCGCT | GCTGATTTGCGTGAGTTCAA | 50 | 178 |
| TM608 | (TGG)5 | TTGAACTCACGCAAATCAGC | CCAGAACCTGAACCCGAAG | 50 | 242 |
| TM609 | (GAG)6 | CATCGATCTCTTCGCAACAA | CAAGCCCCTCTCAAACTCAG | 52 | 191 |
| TM610 | (AAAAT)4 | CTTGCATTCAAGCTCAGCAG | AATATTGTTTGCGCCTTTGC | 52 | 180 |
| TM611 | (TCTTC)3 | AGCACTTGGGGTTGAAATTG | ATCCCCTAACCCCTCATGTT | 52 | 244 |
| TM612 | (TC)14 | CCCTCCAAAACAACGAGAAA | TCGAACCCCATAATTCAAGC | 50 | 270 |

| | | | | | |
|---|---|---|---|---|---|
| TM613 | (ACCCC)3 | GGGAGATCAACAGGTGGTGT | GGAGCACTGGAATGTCCCTA | 52 | 273 |
| TM614 | (CTT)7 | TCTTGACGAACAGATCGACG | CGATTGAAGATAACCCTTTTGG | 52 | 213 |
| TM615 | (TCA)5 | GCAGCCGAAGAAGAAGAATG | AGCCTGAGACAAGGAACAGC | 52 | 204 |
| TM616 | (TC)11 | TGTGTGTGTCTGCGCTACAA | GCCTGGTTCAAATTCATCTACC | 52 | 192 |
| TM617 | (CAACAG)3 | AACAAAAACCGCAAAGATGG | TGTCGGTGGCAACATAGGTA | 52 | 268 |
| TM618 | (TCA)8 | CTGCGATTGCTGTTGTTGTT | TGATCGTGGTCACAGACGTT | 52 | 175 |
| TM619 | (TGTAG)4 | GGGGTTGAGAAATGGGAAAT | GGCAAAACTAGTCCCCTGGT | 52 | 257 |
| TM620 | (CCA)5 | GCCAAAGCAAGATTACCCAA | ATGGGATTTTAGTGGGGGAG | 50 | 145 |
| TM621 | (TAGGGT)3 | CCACTCCTCTCTCTCCATCG | TGCAATGGAACCAACTTGAA | 52 | 278 |
| TM622 | (CTTCT)3 | TCAATTGTCTTCTCATCATAGTCATA | TGAGCAACCAAAGATGCAAG | 52 | 151 |
| TM623 | (CTT)6 | TCCCAATCATCATCATCGAA | GGTTCCCATTGGTTTGTGTC | 52 | 141 |
| TM624 | (AG)9 | TAAATCCCAGGTGTCGATGC | CAGCGTGGTTGTTAGGGTTT | 56 | 114 |
| TM625 | (TA)9 | TGTCAAAAACAAATGAGGCG | TGTGATCGAGCCTTCTGATG | 56 | 257 |
| TM626 | (CAAAC)5 | ATGGGATCTTTTTGCAATGG | GAAGTGTTTGCGTGCACTGT | 54 | 246 |
| TM627 | (CTC)5 | TCAAGCGATTGAAGAACACG | TCAACAAGGCCCAAAGAAAC | 52 | 247 |
| TM628 | (AG)10 | TTCAGCCACAGACACAGACC | TTCCTGAAACCTCTTCATTGG | 54 | 186 |
| TM629 | (GA)10 | CAGAAGAAACCAATCCACCAA | TGCACCCAAACACCTTGTAA | 56 | 192 |
| TM630 | (CAT)6 | CGCCATTGAAATGTGTTGAC | CACCATCTTGCCCTTTTCAT | 52 | 198 |
