# Supplementary material for: Construction of a SSR-Based Genetic Map and Identification of QTLs for Catechins Content in Tea Plant (Camellia sinensis)
Source: PLoS One. 2014 Mar 27;9(3):e93131. doi: 10.1371/journal.pone.0093131 (PMC3968092; doi:10.1371/journal.pone.0093131)
Supplement: Table S2 — Marker names, linkage groups, segregation types, segregation distortion (SD) patterns, and p values for tests of genotypic segregation distortion, for allelic SD in each parent, and for zygotic SD in the ‘YS’×‘BD’ population. (PDF) [file pone.0093131.s005.pdf]

Table S2 Marker names, linkage groups, segregation types, segregation distortion (SD) patterns, and *p* values for tests of genotypic segregation distortion, for allelic SD in each parent, and for zygotic SD in the 'YS' × 'BD' population.

Locus	LG	Segregation type	SD patten[a]	*Pa_mat*[b]	*Pa_pat*[b]	*Pz*[b]
P13	1	nn×np	I		0.021	1
TM325	1	nn×np	I		0.016	1
TM344	1	hk×hk	III	0.284	0.284	0.1
TM345	1	ef×eg	IV	0.002	0.289	0.451
TM503	1	ef×eg	IV	0.454	0.003	0.422
TUGMS102A	1	nn×np	I		0.027	1
TUGMS42	1	nn×np	I		0.03	1
TM622	2	lm×ll	I	0.037		1
TUGMS95	2	nn×np	I		0.015	1
TM051	3	hk×hk	III	0.001	0.001	0.029
TM136	3	nn×np	I		0.001	1
CN02	4	ef×eg	IV	0.044	0	1
TM333	4	nn×np	II		0	1
TM343	4	nn×np	I		0	1
TM369	4	nn×np	I		0	1
TM379	4	lm×ll	I	0.038		1
TM422	4	nn×np	I		0	1
TM432	4	nn×np	I		0	1
TM444	4	ab×cd	IV	0.374	0	0.997
TM445	4	nn×np	I		0	1
TM502	4	nn×np	I		0	1
TM513	4	ef×eg	I	0.037	0	0.972
TM523	4	nn×np	I		0.002	1
TM557	4	nn×np	II		0	1
TUGMS12	4	ef×eg	IV	0.651	0.002	0.879
TUGMS43A	4	ab×cd	IV	0.882	0	0.998
P07	5	nn×np	I		0.009	1
TM105	5	ef×eg	I	0.94	0.029	0.131
TM216	5	ef×eg	I	0.031	0.045	0.968
TM286	5	ef×eg	I	0.542	0.006	0.593
TM349	5	nn×np	I		0.013	1
TM580	5	ef×eg	I	0.053	0.037	0.595
TM162	6	ef×eg	I	0.025	0.099	0.907
TM338	6	lm×ll	I	0.004		1
TM341	6	ef×eg	I	0.023	0.172	0.001
TM169	7	nn×np	II		0.032	1
TM279	7	nn×np	I		0.014	1
TM324	7	nn×np	II		0.045	1
TM367	7	nn×np	I		0.01	1
TM374	7	ef×eg	IV	0.822	0.002	0.572

TM408	7	ef×eg	II	0.414	0.009	0.639
TM415	7	nn×np	I		0.005	1
TM535	7	ef×eg	IV	0.822	0.003	0.663
TM561	7	nn×np	I		0.021	1
TM610	7	nn×np	I		0.014	1
TM217	8	hk×hk	III	0.233	0.233	0.006
TM313	8	hk×hk	IV	0.012	0.012	0
TM352	8	lm×ll	I	0.04		1
TM395	8	lm×ll	I	0.002		1
TM431	8	ef×eg	IV	0.034	0.01	1
TM471	8	lm×ll	I	0.001		1
TM484	8	ef×eg	IV	0.007	0.18	1
TM493	8	lm×ll	I	0		1
TM516	8	lm×ll	I	0.001		1
TM526	8	lm×ll	I	0.017		1
TM550	8	ef×eg	I	0.005	0.151	1
TM578	8	lm×ll	I	0.021		1
TM612	8	ef×eg	I	0.001	0.099	0.841
TM630	8	lm×ll	I	0.004		1
TUGMS51	8	ef×eg	I	0.001	0.17	1
P14	9	ef×eg	III	0.408	0.94	0.035
TM067	9	hk×hk	IV	0.291	0.291	0.087
TM410	9	nn×np	I		0.021	1
TM437	9	nn×np	I		0.011	1
TM541	9	nn×np	I		0.014	1
TM567	9	nn×np	I		0.037	1
TUGMS52	9	ef×eg	III	0.655	0.881	0.03
TM407	10	ef×eg	I	0.054	0.038	0.212
TM458	10	ef×eg	IV	0.072	0.294	0.27
TM475	10	hk×hk	III	0.101	0.101	0.085
TM509	10	ef×eg	I	0.037	0.011	0.182
TM544	10	ef×eg	IV	0.117	0.331	0.176
TM595	10	nn×np	I		0.009	1
TUGMS78	10	ef×eg	I	0.002	0.939	1
TM157	14	nn×np	I		0	1
TM200	14	ef×eg	IV	0.709	0.001	0.508
TM262	14	ef×eg	IV	0.286	0	1
TM285	14	hk×hk	III	0.029	0.029	0.592
TM292	14	nn×np	I		0.002	1
TM330	14	hk×hk	III	0.011	0.011	0.166
TM348	14	lm×ll	II	0.035		1
TM351	14	nn×np	I		0	1
TM464	14	ef×eg	IV	0.368	0.002	0.984
TM483	14	ef×eg	IV	0.499	0	0.989

TM506	14	ef×eg	IV	0.07	0	0.985
TM530	14	nn×np	I		0.001	1
TM553	14	nn×np	I		0	1
TM571	14	nn×np	I		0	1
TM611	14	nn×np	I		0.004	1
TM619	14	nn×np	I		0.001	1
TUGMS23	14	ef×eg	IV	0.23	0	0.978
TM399	15	nn×np	II		0.018	1
TM404	15	ef×eg	II	0.072	0.099	0.303
TM601	15	nn×np	II		0.009	1

[a] Markers exhibiting skewed genotypic frequencies toward 'YS', 'BY', both parents and heterozygote, respectively.

[b] p_{a_mat}, p_{a_pat}, and p_z are the p value of maternal parental allelic SD test, paternal parental allelic SD test, and zygotic SD test, respectively.
