# Supplementary material for: Construction of a SSR-Based Genetic Map and Identification of QTLs for Catechins Content in Tea Plant (Camellia sinensis)
Source: PLoS One. 2014 Mar 27;9(3):e93131. doi: 10.1371/journal.pone.0093131 (PMC3968092; doi:10.1371/journal.pone.0093131)
Supplement: Table S3 — Summary of the significant QTLs (detected in two measurement years) evidenced by both single-QTL model (IM) and restricted multiple QTL model (rMQM) mapping for catechins content using the ‘YS’×‘BD’ tea plant population. (PDF) [file pone.0093131.s006.pdf]

**Table S3 Summary of the significant QTLs (detected in two measurement years) evidenced by both single-QTL model (IM) and restricted multiple QTL model (rMQM) mapping for catechins content using the 'YS' × 'BD' tea plant population.**

| Trait | QTL | LG | Year | Positon (cM ± 2-LOD) | LOD threshold[a] | LOD score | PVE (%) | Nearest marker | a1 [b] | a2 [b] | d [c] |
|---|---|---|---|---|---|---|---|---|---|---|---|
| EC (mg/g) | *qEC3* | 3 | 2010 | 44.0 (37.0-51.7) | 4.2 | 6.39 | 10.5 | TM376 | 4.34 | 0.63 | 1.68 |
| | | | 2011 | 44.7 (41.3-51.7) | 4.3 | 7.88 | 14.5 | TM546 | 4.97 | 2.54 | 1.65 |
| | *qEC11* | 11 | 2010 | 4.8 (3.0-9.8) | 4.2 | 14.86 | 31.2 | TM623 | 7.28 | -4.33 | -2.64 |
| | | | 2011 | 3.0 (0-4.8) | 4.3 | 10.52 | 23.9 | TM586 | 7.15 | -1.89 | -2.48 |
| ECG (mg/g) | *qECG3* | 3 | 2010 | 77.9 (66.2-81.4) | 4.2 | 5.09 | 2.6 | TM560 | -3.14 | 5.55 | 0.34 |
| | | | 2011 | 65.4 (32.0-72.9) | 4.2 | 7.2 | 3.9 | TM453 | -3.42 | 5.87 | 2.46 |
| | *qECG11* | 11 | 2010 | 3.0 (3.0-4.8) | 4.2 | 52.85 | 71 | TM586 | 29.68 | -10.79 | -6.35 |
| | | | 2011 | 3.0 (3.0-4.8) | 4.2 | 55.63 | 69.7 | TM586 | 33.29 | -8.32 | -6.62 |
| | *qECG12* | 12 | 2010 | 64.6 (54.4-67.3) | 4.2 | 4.66 | 2.5 | TM340 | 4.71 | 0.80 | 2.53 |
| | | | 2011 | 59.4 (45.0-67.3) | 4.2 | 4.81 | 2.4 | TM138 | 3.88 | 2.71 | 3.65 |
| | *qECG15* | 15 | 2010 | 13.6 (2.3-13.7) | 4.2 | 7.17 | 3.5 | TM399 | -1.30 | -7.09 | 2.38 |
| | | | 2011 | 13.6 (2.3-19.2) | 4.2 | 7.93 | 3.7 | TM399 | 0.54 | -7.94 | 1.03 |
| EGC (mg/g) | *qEGC2* | 2 | 2010 | - | - | - | - | - | - | - | - |
| | | | 2011 | 7.9 (5.3-8.8) | 4.2 | 9.68 | 15.7 | TM176 | 6.08 | -9.59 | 3.12 |
| | *qEGC3* | 3 | 2010 | 50.5 (41.3-58.0) | 4.3 | 7.25 | 11.9 | TM136 | 16.72 | 1.18 | 1.80 |
| | | | 2011 | 54.4 (49.8-58.0) | 4.2 | 9.26 | 14.9 | TM412 | 11.45 | 4.77 | 2.03 |
| | *qEGC10* | 10 | 2010 | 47.5 (30.3-65.4) | 4.3 | 5.32 | 7.3 | TM407 | -4.86 | -13.51 | 1.47 |
| | | | 2011 | - | - | - | - | - | - | - | - |
| | *qEGC11* | 11 | 2010 | 4.8 (3.0-16.6) | 4.3 | 10.54 | 19.3 | TM623 | -20.30 | -2.25 | -2.89 |
| | | | 2011 | 0 (0-4.8) | 4.2 | 7.21 | 12.3 | TM435 | -10.79 | 1.56 | 3.27 |
| | *qEGC14* | 14 | 2010 | - | - | - | - | - | - | - | - |

| | | | | | | | | | | | |
|---|---|---|---|---|---|---|---|---|---|---|---|
| | | | 2011 | 25.3 (15.0-36.5) | 4.2 | 4.25 | 6.5 | TM200 | 4.80 | 5.59 | -3.08 |
| | *qEGC15* | 15 | 2010 | 41.5 (28.2-52.4) | 4.3 | 4.36 | 5 | TM564 | 8.58 | -8.84 | 5.98 |
| | | | 2011 | - | - | - | - | - | - | - | - |
| EGCG (mg/g) | *qEGCG3-1* | 3 | 2010 | - | - | - | - | - | - | - | - |
| | | | 2011 | 57.8 (32.0-58.0) | 4.3 | 6.37 | 7.3 | TM394 | -13.69 | 10.96 | 1.97 |
| | *qEGCG3-2* | 3 | 2010 | - | - | - | - | - | - | - | - |
| | | | 2011 | 67.1 (65.4-72.9) | 4.3 | 7.24 | 8.6 | TM617 | -16.59 | 6.42 | 0.30 |
| | *qEGCG10* | 10 | 2010 | - | - | - | - | - | - | - | - |
| | | | 2011 | 27.2 (22.2-41.7) | 4.3 | 4.64 | 2.4 | TM433 | 1.28 | 15.15 | -0.40 |
| | *qEGCG11* | 11 | 2010 | 0 (0-4.8) | 4.3 | 17.9 | 40.1 | TM435 | -38.59 | 2.77 | 3.26 |
| | | | 2011 | 0 (0-4.8) | 4.3 | 29.13 | 52.4 | TM586 | -42.67 | 3.78 | 3.64 |

*EC* epicatechin, *ECG* epicatechin gallate, *EGC* epigallocatechin, *EGCG* epigallocatechin gallate, *LOD* logarithm of odds ratio, *PVE* phenotypic variation explained

[a] The genome-wide LOD significance thresholds ($P < 0.05$) based on permutation testing (n=1000)

[b] a1and a2 represent the additive (or average allele substitution) effects from maternal parent and paternal parent, respectively.

[c] The overall dominance effects
