# Supplementary material for: Construction of a SSR-Based Genetic Map and Identification of QTLs for Catechins Content in Tea Plant (Camellia sinensis)
Source: PLoS One. 2014 Mar 27;9(3):e93131. doi: 10.1371/journal.pone.0093131 (PMC3968092; doi:10.1371/journal.pone.0093131)
Supplement: Table S4 — Summary of primer screening of the novel genic SSRs based on PCR amplification. DNA from the mapping parents was used to verify the polymorphisms of the SSR primers. (PDF) [file pone.0093131.s007.pdf]

Table S4 Summary of primer screening of the novel SSRs based on PCR amplification. DNA from the mapping parents, ‘YS’ and ‘BD’, was used to determine the polymorphisms of the SSR primers.

Type	Aelle	No. of primers	Proportion (%)
Failed	0	310	27
Monomorphic	1	361	32
Polymorphic[1]	2	142	12
Polymorphic[2]	2/3/4	328	29

[1] Primers yield two homozygous alleles.

[2] Primers yield at least one heterozygous allele.
